# Noninvasive malaria detection beyond blood sampling

**DOI:** 10.1039/d6sd00085a

**Published:** 2026-07-21

**Authors:** Shaun G. Hong, Jung Woo Leem, Haripriya Sakthivel, Semin Kwon, Sang Mok Park, Atin Dewan, David J. Botana, Young L. Kim

**Affiliations:** a Weldon School of Biomedical Engineering, Purdue University West Lafayette Indiana 47907 USA; b Charles Stark Draper Laboratory Cambridge Massachusetts 02139 USA; c Department of Intelligent Systems Engineering, Indiana University Bloomington Indiana 47408 USA kimyl@iu.edu; d Department of Radiology and Imaging Sciences, Indiana University School of Medicine Indianapolis Indiana 46202 USA; e Center for Global Health, Indiana University Indianapolis Indiana 46202 USA

## Abstract

Malaria remains a major global health challenge, particularly in sub-Saharan Africa, despite being one of the oldest documented human diseases. Although blood-based detection remains the mainstream diagnostic modality, its use is constrained by the need for trained personnel, sterile procedures, patient discomfort, and reduced sensitivity for low-parasitemia and asymptomatic infections. Recent advances in sensing and diagnostics, increasingly integrated with machine learning and artificial intelligence, have created new opportunities for noninvasive malaria detection using accessible biological samples and physiological signals. This critical review synthesizes recent progress in noninvasive malaria detection across nonblood sampling matrices, biophysical detection mechanisms, and data-driven analytical approaches. We examine diverse sampling matrices, including urine, saliva, exhaled breath, and skin-emitted volatile organic compounds with attention to their associated biomarkers, biological relevance, and operational feasibility. We further categorize detection technologies into three principal domains: biological sample-based platforms, including immunoassays, molecular amplification, biosensors, and mass spectrometry; acoustic, photoacoustic, and optical systems, including ultrasound, photoacoustics, optical imaging, and spectroscopy; and digital and connected health platforms, including wearable monitoring, smartphone imaging, and machine learning-enabled analysis. We then compare these modalities in terms of analytical performance, operational suitability, and translational potential for integration into healthcare systems. Finally, we present an implementation-oriented roadmap that highlights translational, equity, artificial intelligence, scalability, and regulatory considerations, identifying practical paths toward field-ready, affordable, and programmatically relevant noninvasive malaria detection.

## Abstract 2

This review maps noninvasive malaria detection without blood sampling, highlighting easily accessible sample matrices and advanced detection mechanisms, including artificial intelligence, that can support malaria control and elimination. Image created with ChatGPT Enterprise.

## Graphical abstract

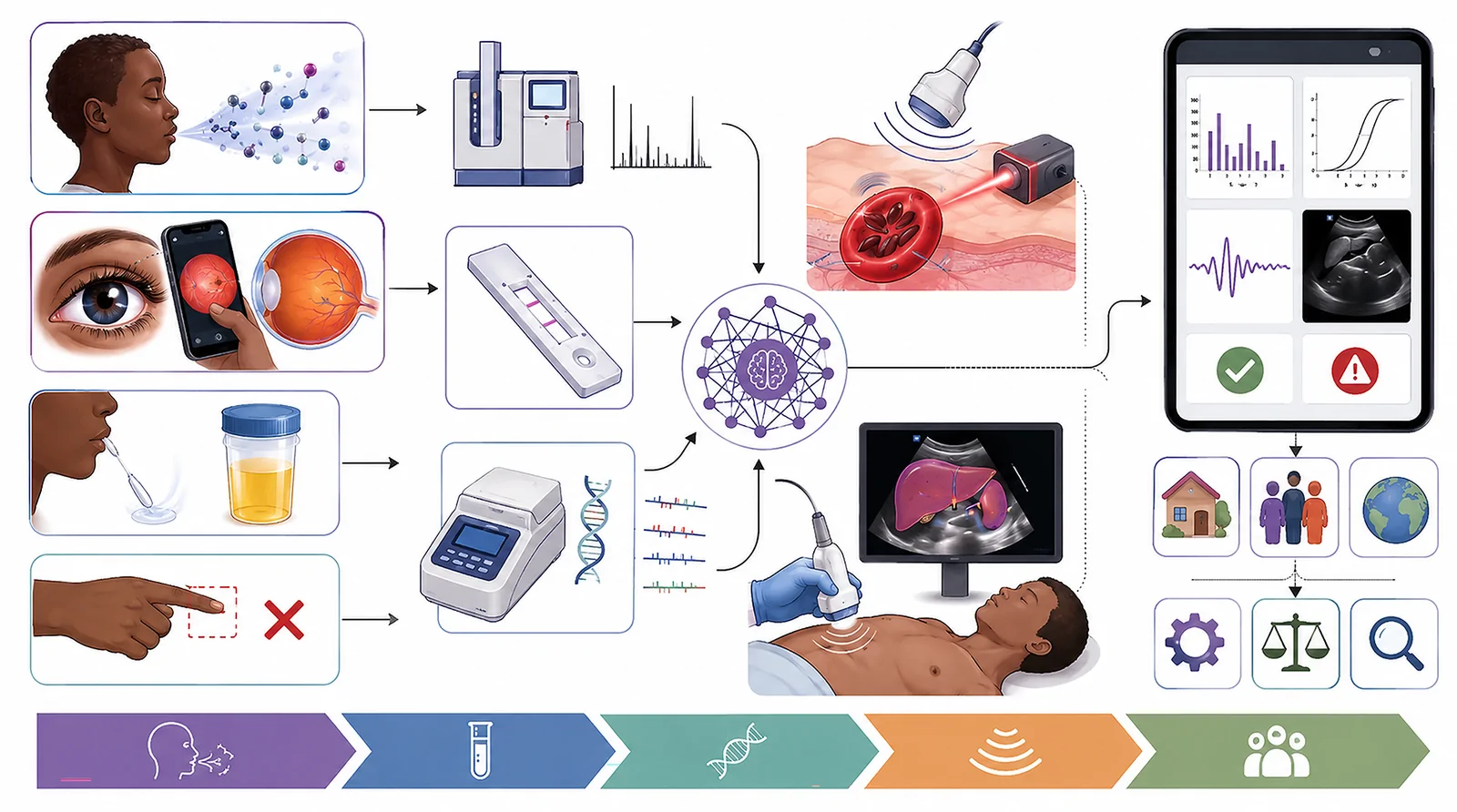

## 1. Introduction

### 1.1. Global burden

Malaria remains a major global health challenge, causing approximately 263 million cases and 597 000 deaths worldwide in 2023, with sub-Saharan Africa accounting for over 94% of the burden.^1–3^ Although 18 countries have been certified malaria-free since 2000, the recent surge in malaria prevalence is driven by multiple factors, including increased resistance to treatments, climate variability, and persistent reservoirs of asymptomatic human infections.^2,4–8^ The urgency for equitable diagnostic solutions is further underscored by recent warnings that malaria is poised for a devastating comeback in Africa due to funding gaps and biological threats.^9^ Current vaccines, including RTS,S/AS01 and R21/Matrix-M, are important additions to malaria control but provide partial protection and require multi-dose delivery and cold-chain implementation, which remain challenging in remote or resource-limited settings.^10,11^ The World Health Organization has warned of increasing resistance to antimalarial drugs and vector control, as well as growing transmission in urban settings, which together threaten to reverse decades of progress.^1,6^

Malaria results from infection with protozoan parasites of the genus *Plasmodium*, which are transmitted to humans through the bite of female *Anopheles* mosquitoes.^15^ Among approximately 156 *Plasmodium* species that infect vertebrates, five are known to infect humans: *Plasmodium falciparum* (*P. falciparum*), *Plasmodium malariae*, *Plasmodium vivax* (*P. vivax*), *Plasmodium ovale* (*P. ovale*), and *Plasmodium knowlesi*.^2,16,17^ Among these, *P. falciparum* is the most dangerous and can be life-threatening, as severe infections may lead to complications including liver and kidney failure, seizures, and coma. In contrast, *P. vivax* and *P. ovale* infections are usually less severe, but they can remain dormant in the liver for extended periods, causing relapses and recurrent symptoms months or even years after the initial illness. From a diagnostic perspective, this diversity of species, life-cycle stages, and parasite densities presents a major challenge: detection platforms must provide high analytical sensitivity while preserving species-level discrimination. If all species cannot be differentiated, distinguishing *P. falciparum* from non-falciparum infections is often beneficial in sub-Saharan Africa. Such capability is essential for guiding appropriate treatment, identifying mixed infections and relapse-prone cases, and supporting surveillance.

### 1.2. Conventional blood-based diagnosis and challenges

Efforts to achieve a malaria-free world follow a continuum from control to pre-elimination, elimination, and finally prevention of reintroduction.^12,13^ Malaria detection and diagnosis play a key role in all malaria programs. Malaria control emphasizes accurate, timely, affordable, and field-deployable diagnosis for routine symptomatic case management. Malaria elimination requires these same attributes plus the ability to detect low-density, asymptomatic, and residual infections and to support surveillance, high-throughput screening, and outbreak response. Achieving malaria control, elimination, and eradication depends on testing all suspected and asymptomatic infections,^4,5,14^ which highlights the urgent need for affordable, scalable, and especially noninvasive detection tools. Conventional malaria diagnosis relies on rapid diagnostic tests (RDTs), microscopy of blood smears, and nucleic acid amplification techniques, including polymerase chain reaction (PCR) and reverse transcription loop-mediated isothermal amplification (RT-LAMP).^18–20^

WHO recommends prompt parasite-based diagnosis by quality-assured light microscopy or RDTs for all patients with suspected malaria before treatment. WHO-prequalified RDTs are assessed against minimum quality and performance requirements and are considered suitable for procurement and clinical malaria diagnosis. More than 120 malaria RDT brands, produced by approximately 60 companies, are currently available, with substantial variation in diagnostic performance, manufacturing quality, and price.^154^ Molecular assays, including PCR and other nucleic acid amplification tests, provide greater sensitivity for low-density infections but are primarily used for reference testing and elimination settings rather than routine field diagnosis.

Malaria RDTs detect parasite antigens in a finger-prick blood sample using antibodies embedded in a lateral-flow strip. The most common target, *P. falciparum* histidine-rich protein 2 (PfHRP2), is abundant and highly sensitive for detecting *P. falciparum* infection. Other RDT targets include *Plasmodium* lactate dehydrogenase (pLDH), available in *P. falciparum*-specific, *P. vivax*-specific, or pan-*Plasmodium* formats, and aldolase, a pan-*Plasmodium* antigen that supports detection of multiple human malaria species. Combination RDTs that detect both species-specific and pan-malaria antigens can improve species differentiation and reduce reliance on a single biomarker. However, RDTs may fail to detect low-parasitemia infections. PfHRP2 may persist after parasite clearance, producing positive results after successful treatment. More importantly, *P. falciparum* strains with histidine-rich protein *pfhrp2* and *pfhrp3* (*pfhrp2/3*) gene deletions can evade PfHRP2-based tests.^21,25,26^

Malaria microscopy examines Giemsa-stained thick and thin blood smears under a light microscope to directly identify *Plasmodium* parasites. In a thick smear, red blood cells are lysed during staining, concentrating parasites in a smaller area and improving sensitivity for detecting low-density infections. In a thin smear, red blood cells remain intact, allowing the microscopist to identify the *Plasmodium* species, recognize parasite stages, and estimate parasitemia as the percentage of infected red blood cells. Together, thick and thin smears provide parasite detection, species identification, and quantitative assessment, making microscopy the traditional reference method for malaria diagnosis. However, accuracy depends heavily on specimen quality, staining, microscope access, and experienced microscopists, and examination can be labor-intensive and less reliable at very low parasite densities.

### 1.3. Noninvasive malaria detection landscape

Noninvasive detection methods that reduce the need for blood sampling, such as finger-prick or venipuncture, have long been of interest. Blood sampling faces operational, acceptability, and scalability challenges in real-world settings. Blood collection requires trained personnel, sterile tools, and appropriate biosafety protocols, which are often unavailable in remote or low-resource settings.^7,15,21^ The invasive nature of blood sampling can be problematic for children, individuals with needle phobia, or communities with cultural resistance, and may occasionally result in hemolysis or insufficient volume, compromising test accuracy.^22–24^ Thus, noninvasive malaria detection methods use alternative, easily accessible biological samples and peripheral tissue sites, including urine,^27–50^ saliva,^27,29,31–36,38,39,41,44,45,51–64^ buccal mucosa,^34,36,65–67^ exhaled breath,^68–73^ and body sites such as skin, eye, ear, and hair.^69,73–94^

Depending on the sample type, a variety of noninvasive detection methods have emerged, including fluid-based immunoassays, such as HRP2 lateral flow,^30,37,40,42–47,52,53,61^ saliva- or urine-based PCR and RT-LAMP,^31–36,38,39,41,56–58,62,87^ electrochemical or nanoparticle biosensors,^54,55,59,60,63^ volatile organic compound profiling from breath using gas chromatography-mass spectrometry (GC-MS),^71,72,74,75^ acoustic ultrasound,^76,78–80,82,84,85,89,90^ photoacoustic detection of hemozoin,^81,83,92^ optical and vibrational spectroscopy,^48,67,73,77^ wearable physiological monitoring,^86,88,91^ and mobile imaging.^94^ Digital and connected health platforms combined with machine-learning-powered analytics can process multimodal physiological data, such as heart rate and fever dynamics, or conjunctiva images for automated interpretation and early, decentralized detection, particularly in pediatric and resource-limited contexts. In addition, experimental platforms using animal models and laboratory simulations are essential for validating sensing mechanisms, optimizing assay interfaces, and guiding the translation of malaria detection toward field-deployable applications.^95–103^

### 1.4. Rationale and scope of this review

Recent advances in biosensing, molecular diagnostics, spectroscopy, mobile health, wearable sensing, and artificial intelligence have expanded the noninvasive malaria detection landscape. However, the field remains fragmented because many studies emphasize individual technologies rather than comparing how each approach aligns with programmatic needs across the malaria control-to-elimination continuum. This review evaluates noninvasive detection not only by technical novelty, but also by biological plausibility, analytical performance, operational feasibility, equity, scalability, and regulatory translation.

We first examine nonblood sampling matrices in section 2, including urine, saliva, buccal mucosa, exhaled breath, skin-emitted VOCs, and other body-derived sources, with emphasis on biomarker origin, diagnostic relevance, and sampling practicality. In section 3, we organize detection methods into three categories: 1) nonblood biological sample-based methods, including immunoassay-based strips, nucleic acid amplification, biosensors, and GC-MS; 2) acoustic, photoacoustic, and optical systems, including ultrasound, photoacoustics, and spectroscopy or imaging; and 3) digital and connected platforms that combine wearable devices, mobile imaging, and AI-enabled analytics. Section 4 then compares these technologies across implementation dimensions and discusses their context-specific utility, limitations, equity implications, AI considerations, scalability, and regulatory pathways. Overall, this review is intended to provide a practical roadmap for moving noninvasive malaria detection beyond proof-of-concept studies toward field-ready and programmatically useful applications.

## 2. Nonblood sampling matrices for noninvasive malaria detection

**Fig. 1:**
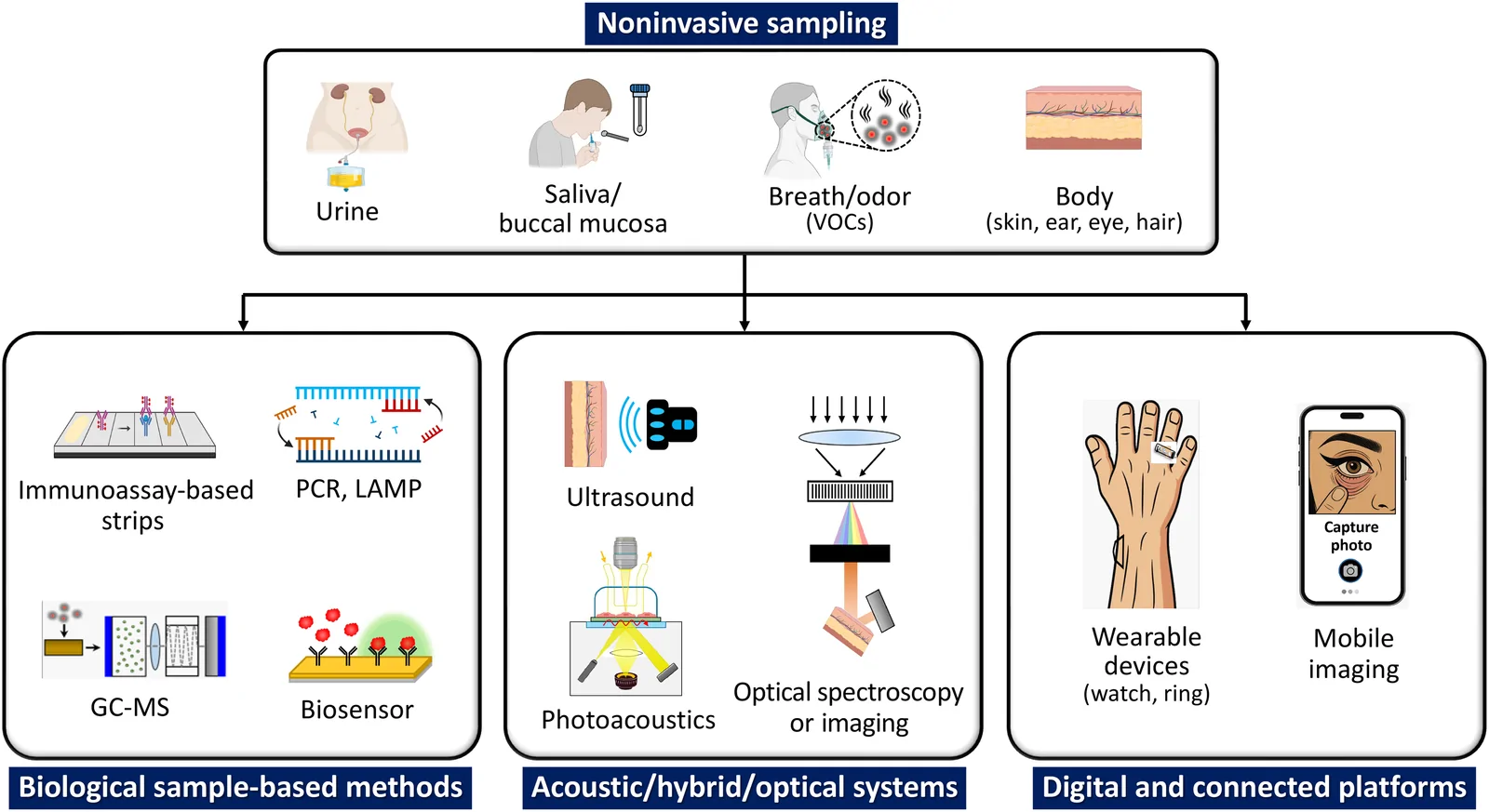
Overview of noninvasive sampling matrices and detection mechanisms for malaria. Illustrated are noninvasive biological sources including urine, saliva, buccal mucosa, exhaled breath, skin-emitted odors, and body-derived sites (*e.g.*, skin, ear, eye, and hair). These matrices provide parasite- or host-derived indicators and support three categories of detection modalities. Biological sample-based methods detect antigens, nucleic acids, antibodies, or volatile organic compounds (VOCs) from urine, saliva, buccal swabs, and breath using immunoassay-based strips, nucleic acid amplification (*e.g.*, polymerase chain reaction (PCR), loop-mediated isothermal amplification (LAMP)), biosensors, or gas chromatography-mass spectrometry (GC-MS). Acoustic, photoacoustic, and optical systems capture transdermal, organ-level, or pigment-related signals using ultrasound, photoacoustics, and optical spectroscopy or imaging without requiring fluid sampling. Digital and connected health platforms employ wearable devices and mobile imaging enhanced with AI-powered analytics to monitor physiological or visual indicators, enabling real-time, noninvasive malaria detection in field settings. Original illustration created by the authors in part using https://BioRender.com and ChatGPT.

Nonblood sampling matrices, including urine, saliva, buccal mucosa, exhaled breath, skin-emitted odors, and other body-derived sources (*e.g.*, skin, ear, eye, and hair), offer accessible and patient-friendly avenues for noninvasive malaria detection (Fig. 1 and Table 1). These matrices encompass a range of biomarkers and physiologically relevant indicators, including parasite antigens, nucleic acids, host immune components, volatile organic compounds (VOCs), and functional responses (*e.g.*, skin conductance or temperature changes, heart rate, and activity levels). Each sampling type presents unique opportunities and challenges in terms of biomarker concentration, stability, and ease of acquisition. This section outlines the major biomarkers accessible from each nonblood sampling matrix and examines the available detection evidence, biological validity, and practical feasibility for malaria in both clinical and field applications, along with key implementation considerations.

**Table 1:** Comparison of noninvasive malaria sampling matrices

Sample matrix	Molecular and physiological markers	Detection techniques	Advantages	Limitations
Urine	PfHRP2 (ref. 30, 37, 40, 42–44, 46, 47 and 49)	Immunoassay-based strips/urine malaria test (UMT)^30,37,40,42,43,46,47,49^	Easy to collect, painless	Low and variable biomarker concentration with lower sensitivity than blood
	pLDH^40,44,47^	Polymerase chain reaction (PCR)/loop-mediated isothermal amplification (LAMP)/nucleic acid sequence-based amplification (NASBA)^31–35,38,39,41,44,45^	No need for trained phlebotomists or sharps	Biomarker instability from pH, enzymatic activity, or storage conditions
	Deoxyribonucleic acid (DNA) (cytochrome b (*cytb*), 18S ribosomal ribonucleic acid (rRNA), mitochondrial DNA)^31–36,38,39,41,45^	Spectroscopy/mass spectrometry^48,50^	Culturally acceptable	Influence of collection timing and sample handling
	Hepcidin and metabolites (cystine, tyrosine (Tyr), lysine (Lys), histidine (His), creatinine (Crn))^48^		Safe for repeat sampling	Variability from hydration status, renal function, and circadian rhythms
			Feasible for large-scale field use	
Saliva	PfHRP2 (ref. 44, 45, 52, 55, 60, 61 and 64)	Immunoassay-based strips^44,45,61^	Painless, repeatable sampling	Low biomarker concentration
	pLDH^44,53^	PCR^31–36,39,41,44,45,56–58,62,63^	Supports both molecular and protein targets	Variability due to hydration, oral health, and diet
	18S ribosomal ribonucleic acid (18S rRNA)/nuclear DNA^31–35,38,39,41,44,45,56–58,62,63^	LAMP^38^	Noninvasive, suitable for pediatric and community screening	Reduced sensitivity in low-density or asymptomatic infections
	Cytochrome b (*cytb*), cytochrome c oxidase subunit 3 (*cox3*), var gene acidic terminal sequence (*varATS*)^35,39,58,62^	Enzyme-linked immunosorbent assay (ELISA)^52–55,64^		Lack of standardized collection protocols
	Gametocyte-specific transcripts (*P. falciparum* vacuole membrane protein (Pfs16)-, *P. falciparum* surface protein 25 (Pfs25)-messenger ribonucleic acid (mRNA))^36^	Biosensors (enzyme activity)^59^		
	Anti-merozoite surface protein 1 (MSP1), anti-circumsporozoite protein (CSP), anti-*P. falciparum* erythrocyte membrane protein 1 (PfEMP1) immunoglobulin G (IgG)^54^	Immunosensors^60^		
	Topoisomerase I^59^			
Buccal mucosa	DNA: 18S rRNA, MSP^34,36^	PCR/droplet digital PCR (ddPCR)^34,36^	Simple, safe, minimal invasiveness	Low sensitivity (especially in low-parasitemia or asymptomatic cases)
	RNA: Pfs16 (gametocyte)^36^	Quantitative NASBA (QT-NASBA)^36^	Direct access to superficial vasculature	RNA instability
	Hemozoin^65–67^	Optical imaging^65–67^	Needle-free, swab-based collection	Variable yield due to collection method differences
VOCs (breath/skin)	Thioethers: allyl methyl sulfide, 1-methylthio-propane, (*E*/*Z*)-1-methylthio-1-propene^72,74,75^	Gas chromatography-mass spectrometry (GC-MS)^71,72,74,75^	Contact-free and noninvasive sampling	Strongly influenced by diet and environment
	Terpenes (plant-derived): α-pinene, 3-carene^72,74,75^		Repeatable testing without sample depletion	Lack of standardized collection and analysis protocols
	Branched alkanes: methyl undecane, dimethyl decane, trimethyl hexane^71,72,75^		No consumables or complex logistics	Variable and low biomarker concentrations
	Aldehydes: nonanal, hexanal, heptanal, octanal, decanal^71,72,74,75^			Limited understanding of biological origin of VOC signatures
	Ketones: 6-methyl-5-hepten-2-one^72,74^			
	Aromatic hydrocarbons: toluene^72^			
	Isoprene^71,72^			
Body-derived sources (skin, ear, eye, hair)	Organ changes (liver, spleen, kidney, lung, brain)^76,78–80,82,84,85,89,90,93^	PCR^87^	Multiparametric and continuous monitoring	Low specificity due to nonspecific symptoms
	Hemozoin^81,83,92^	Ultrasound imaging^76,78–80,82,84,85,89,90,93^	Noninvasive, painless, and field-deployable	Device dependency and environmental sensitivity
	Skin physiological signals (temperature, perfusion, conductance)^77,86,88,91^	Optical & photoacoustic techniques^77,81,83,92^	Minimal biosafety risk, no sharps or hazardous waste	Limited standardization and large-scale validation
	Hair DNA (*P. falciparum* DNA)^87^	Wearable sensors^86,88,91^		Inter-individual and temporal variability (*e.g.*, physiological and circadian effects)
	Systemic responses (heart rate, temperature, gastrointestinal activity)^88,91^	NIR spectroscopy (NIRS)^73^		
	Near-infrared (NIR) optical skin absorption^73^	Mobile imaging (smartphone)^94^		
	Eye (inner eyelid, conjunctiva)^94^			

### 2.1. Urine

Urine is a noninvasive and easily accessible sample that can be self- or community-collected without trained personnel, offering practical advantages for field screening and repeated surveillance.^27–29^ Reported urine biomarkers can be grouped by biological origin and clearance route. Parasite antigens such as PfHRP2 and pLDH are released during blood-stage infection and may appear in urine after renal filtration of soluble antigens or antigen–antibody complexes, making them indicators of active or recent parasitemia.^15,30,37,40,42–44,46,47,49,50^ Parasite nucleic acids, including mitochondrial *cytb* and 18S rRNA targets, are typically present at trace levels and may enter urine during parasite breakdown, renal passage, or immune clearance; amplification is usually required for species-level detection.^31–36,38,39,41,44,45^ Host-response metabolites, including cystine, tyrosine, lysine, histidine, and creatinine, reflect infection-associated metabolic perturbation rather than direct parasite products.^48^ Proteomic studies have also identified parasite-associated proteins such as rifin-like protein, profilin, Duffy binding-like merozoite surface protein 2, and single-stranded DNA-binding protein in urine, supporting broader biomarker discovery for noninvasive assays.^50^

Urine-based detection methods include lateral flow immunoassay-based strips,^30,37,40,42,43,46,47^ PCR or related nucleic acid amplification approaches,^31–35,39,41,44,45^ and metabolite or proteomic profiling with spectroscopy or mass spectrometry.^48,50^ Collectively, these studies demonstrate feasibility but also show that performance depends strongly on target abundance, parasite density, sample handling, and assay chemistry. Compared with blood, urine generally contains lower and more variable concentrations of parasite-derived material, which reduces sensitivity, particularly in low-density or asymptomatic infections.^31,33,38,40,42,44^

Reliability will require both improved biomarkers and practical normalization strategies. Hydration status, renal function, urine concentration, and circadian variation can dilute or concentrate urinary biomarkers independently of infection status.^104–106^ Future studies should consider creatinine normalization, specific-gravity correction, standardized sampling time, internal process controls, longitudinal within-person monitoring, and multi-biomarker panels to reduce dilution and physiological variability. These design choices are especially important if urine-based tests are used for community surveillance, where collection conditions may differ substantially across households, schools, and clinics.

### 2.2. Saliva

Saliva is a readily accessible biological fluid that can be collected without trained personnel, making it suitable for pediatric, field, and repeated-sampling applications.^15,29,51^ Salivary biomarkers can be organized into complementary categories. Parasite antigens such as PfHRP2 and pLDH indicate active or recent infection and have been targeted by ELISA, lateral flow assays, and electrochemical or impedimetric immunosensors.^44,45,52–55,60,61,64^ Parasite-derived enzymes, including aldolase, *Plasmodium* glutamate dehydrogenase (pGDH), and topoisomerase I, reflect parasite metabolic or enzymatic activity and support enzyme-based biosensor readouts.^53,59^ pGDH is soluble, relatively heat-stable, and genetically conserved in *Plasmodium*, which may support pan-malaria or *P. falciparum* detection, although it remains an emerging biomarker.^53^ Parasite DNA targets, including 18S rRNA, *cytb*, *cox3*, and *varATS*, provide species-level molecular evidence, with multicopy mitochondrial or repetitive targets improving detectability in low-density infections.^31–35,38,39,41,44,45,56–58,62,63^ Gametocyte-specific transcripts such as Pfs16 and Pfs25 mRNA have been explored as markers of transmission reservoirs, whereas host antibodies to MSP1, CSP, and PfEMP1 reflect immune exposure and may complement antigen or nucleic acid detection.^36,54^

Analytical platforms should be interpreted in relation to the biomarker class they target. Immunoassays and immunosensors are operationally simple but may be limited by low antigen concentration in saliva. Molecular methods, including nested PCR, quantitative PCR, ddPCR, and LAMP, can improve sensitivity and species discrimination but require careful sample preparation and amplification controls. Enzyme-activity and integrated biosensor systems offer routes toward rapid, label-free detection, shorter assay times, and deployment in resource-limited settings, but still require validation in larger clinical and field cohorts.^31–36,39,41,44,45,56–60,62,63^

Several challenges continue to limit saliva-based assays. Biomarker concentrations are typically lower than in blood, and salivary composition can vary with hydration status, oral health, diet, circadian timing, and collection method, reducing consistency near the detection limit.^41,43,45,51,106–108^ These sources of variation should be addressed through standardized collection protocols, including consistent sampling time, pre-collection restrictions when feasible, sample handling, sample-volume normalization, internal controls, paired biomarker panels, and, when available, normalization to a validated salivary reference marker rather than through biomarker discovery alone. Lack of standardized saliva collection and processing protocols also continues to hinder cross-study comparisons and reproducibility. Despite these limitations, saliva remains attractive because it is safe, repeatable, broadly acceptable, and well suited to community-based or longitudinal surveillance, provided that adherence and context-specific sampling practices are incorporated into study design.^44,45,54,61,109^

### 2.3. Buccal mucosa

The buccal mucosa, the inner lining of the cheek, is an accessible and noninvasive site for malaria detection, offering a swab-based collection method that avoids needles, biohazardous waste, or specialized training.^27,34,36^ The superficial vasculature and thin epithelium of the buccal mucosa make it particularly suitable for detecting parasite-derived nucleic acids, including *Plasmodium* DNA and RNA,^34,36^ as well as endogenous or structural signals, including flowing blood cells and tissue microarchitecture.^65,66^ Hemozoin, a crystalline pigment formed by *Plasmodium* during the digestion of hemoglobin, accumulates within infected red blood cells and can circulate through the microvasculature, including vessels near the mucosal surface. This positions the buccal region as an accessible window for visualizing parasitemia-related features, including endothelial glycocalyx disruption and perivascular hemorrhages observed in pediatric malaria^67^ that may not be evident in secretions alone.

Several detection methods using the buccal mucosa have been investigated, ranging from PCR-based assays that detect parasite DNA, including 18S rRNA and MSP genes,^34,36^ to quantitative nucleic acid sequence-based amplification (QT-NASBA) amplification of gametocyte-specific transcripts (*e.g.*, Pfs16).^36^ Optical techniques, including reflectance confocal microscopy,^65^ spectrally encoded flow cytometry,^66^ and *in vivo* mucosal imaging,^67^ have been applied to visualize hemozoin and microvascular alterations. Together, these studies support buccal swabs as a needle-free source of parasite nucleic acids and mucosal imaging as a real-time window into malaria pathophysiology.^34,67^ However, buccal sampling generally yields lower sensitivity than blood or saliva, due to the low abundance of biomarkers, RNA degradation, and variability in collection methods.^34,36^ In addition, variability in cellular yield, mucosal integrity, and swab collection depth influence biomarker recovery, contributing to inter-sample variability, particularly in low-density infections.^51,68^ Even with these limitations, its ease of collection, safety, and compatibility with portable molecular and optical platforms support its role as a complementary approach for noninvasive malaria detection.

### 2.4. Volatile organic compounds

Volatile organic compounds (VOCs) are gaseous metabolites released from breath and skin whose profiles can shift with infection-associated changes in host and parasite metabolism.^70^ In malaria, reported VOC signatures include thioethers, branched alkanes, aldehydes, ketones, aromatic hydrocarbons, isoprene, and terpenes, with representative compounds summarized in Table 1 and Table 3 rather than repeated extensively in the main text.^71,72,74,75^ Mechanistically, branched alkanes and aldehydes may reflect oxidative stress and lipid peroxidation, whereas terpene-related signals may be linked to altered isoprenoid metabolism or mosquito-attraction biology.^71,72,74,75^

The diagnostic relevance of VOCs is supported by both breath and skin studies. Breath studies have identified malaria-associated thioether or mixed VOC panels that distinguish infected from uninfected participants, whereas skin-emitted VOC studies in malaria-endemic settings have reported predictive signatures in symptomatic, asymptomatic, and submicroscopic infections.^71,72,74,75^ Importantly, some VOC panels were evaluated against non-malarial febrile illness, suggesting that chemical patterns may provide information beyond nonspecific fever alone.^72,74,75^

Despite these findings, VOC detection remains highly sensitive to diet, age, environment, co-infections, sampling material, storage conditions, and analytical workflow.^68,70–72,74,75,105,110^ Many candidate compounds occur at low and variable abundance, and the biological pathways producing malaria-associated VOCs remain incompletely defined. Translation will require standardized breath or skin-odor collection, quality-control compounds or environmental blanks, harmonized GC-MS or sensor-array processing, and external validation before VOC profiling can move from biomarker discovery toward field-deployable electronic-nose or AI-enabled sensing systems.^70^

### 2.5. Body-derived sources

The human body provides multiple noninvasive sites that reflect anatomical and physiological changes during malaria infection. Abdominal ultrasonography can assess hepatosplenic and renal changes,^76,78,80,82,93^ while pulmonary imaging reveals malaria-associated lung pathology.^85,89^ In cerebral malaria, raised intracranial pressure is inferred through the optic nerve sheath diameter (ONSD) or cerebral blood flow velocity measured by transcranial Doppler ultrasound.^79,84,90^ Ocular sites, including the conjunctiva, are also emerging as accessible windows to visualize microvascular alterations without interference from skin pigmentation, because malaria causes changes to microvascular properties and dynamics.^94^

As the largest organ, the skin offers another accessible surface for noninvasive malaria detection. Physiological signals can be obtained from peripheral sites, including the fingers, wrists, earlobes, and forehead, where inflammatory responses may alter temperature, perfusion, or conductance.^77,86,88,91^ Hair has also been investigated as a matrix for parasite DNA or metabolic byproducts.^87^ Wearable devices further enable continuous monitoring of systemic responses, including heart rate, temperature, and gastrointestinal activity, providing complementary host–parasite information in field settings.^88,91^ Optical and photoacoustic methods applied through the skin, including vapor nanobubble-based transdermal detection,^81^ portable prototype systems integrating optical and acoustic sensing,^83^ and *in vivo* photoacoustic detection of hemozoin,^92^ have expanded the range of measurable signals. Near-infrared spectroscopy has further enabled rapid, reagent-free detection through the skin.^103^

Body-derived sources often face inherent limitations in biomarker specificity. Physiological alterations such as fever, anemia, tachycardia, altered perfusion, splenomegaly, or pulmonary edema are common to many infectious and inflammatory diseases, complicating attribution to malaria alone. These signals are also shaped by host condition, circadian rhythm, disease stage, and treatment timing.^105,106,111^ Imaging and instrumentation-based approaches add further implementation constraints: ultrasound can be portable but is operator-dependent and requires training, calibration, maintenance, and reliable power; photoacoustic systems require laser safety controls, optical alignment, and specialized detectors; and optical spectroscopy can be affected by skin pigmentation, motion, probe placement, and device calibration. These requirements may limit feasibility for large-scale rural screening in remote malaria-endemic villages unless devices are simplified, ruggedized, and paired with practical training and quality-assurance workflows.

Nevertheless, body-derived indicators are easily measurable, well tolerated, and suitable for longitudinal surveillance when interpreted as screening, triage, severity assessment, or risk-stratification signals rather than stand-alone confirmatory diagnostics. Integration with wearable devices,^88,91^ mobile imaging platforms,^94^ and AI-driven analytics^91,94^ may improve noninvasive screening and access in settings where conventional blood-based testing is less feasible, provided that results are validated against appropriate parasitological reference standards.

### 2.6. Additional considerations for nonblood sampling matrices in noninvasive malaria detection

Across these nonblood sampling matrices, successful implementation depends not only on analytical performance but also on user acceptability, collection consistency, environmental conditions, storage and transport requirements, and access to processing infrastructure.^6^ These factors are especially important in malaria-endemic regions where testing may occur through community health workers, schools, household surveillance, antenatal programs, or mobile outreach rather than conventional laboratories. Field examples illustrate the point: saliva-based Plasmodium sexual stage protein 17 (PSSP17) testing has been evaluated in asymptomatic children in Cameroon and Zambia and in febrile patients in Sierra Leone,^61^ smartphone conjunctiva imaging has been studied for malaria risk stratification in asymptomatic school-age children,^94^ and user-centered studies in Peru, Indonesia, and Rwanda highlight that blood-free testing can be attractive but still depends on trust, privacy, instructions for self-collection, and confidence in test accuracy.^109,157^

Noninvasive matrices such as urine and saliva may be particularly useful for women, children, and caregivers when privacy, bodily integrity, fear of needles, or repeated testing are major barriers.^25,109,157^ However, their deployment should be designed around local workflows, including clear collection instructions, culturally acceptable sample handling, mechanisms for returning results, and linkage to confirmatory testing or treatment. Community engagement should be treated as an implementation requirement rather than a generic equity statement, particularly in resource-limited, geographically isolated, or school- and household-based surveillance contexts.

## 3. Methods for noninvasive malaria detection

This section classifies noninvasive malaria detection methods into three domains based on sampling strategy and detection modality (Fig. 1). First, we examine nonblood biological sample-based detection methods, which identify infection-related biomarkers in urine, saliva, buccal mucosa, hair, and breath/skin odor using immunoassay-based strips; nucleic acid amplification, such as polymerase chain reaction (PCR), loop-mediated isothermal amplification (LAMP), or nucleic acid sequence-based amplification (NASBA); biosensors; and gas chromatography-mass spectrometry (GC-MS). Second, we distinguish acoustic ultrasound, photoacoustic methods, and optical spectroscopy or imaging because these modalities differ in their physical signal origin, instrumentation, and interpretation. Finally, we discuss digital and connected platforms for malaria detection, highlighting recent advances in wearable sensors, mobile imaging, and AI-powered analytics for interpreting physiological signals or visual cues in a noninvasive manner.

### 3.1. Nonblood biological sample-based detection methods

A range of detection methods has been developed to identify malaria from nonblood samples, building on biomarkers included in urine, saliva, buccal mucosa, hair, and breath or skin odor. This section focuses on four representative platforms, including immunoassay-based strips, nucleic acid amplification (*e.g.*, PCR, LAMP, NASBA), biosensors, and GC-MS, and reviews their applications in noninvasive sample matrices with respect to both analytical performance and practical feasibility (Table 2).

**Table 2:** Summary of studies on nonblood biological sample-based detection methods for malaria

Detection method	Sample type	Target biomarker	Platform details	Performance (sensitivity/specificity/accuracy)	Detection time	Low-resource field usability	Research population	Research area	Detect asymptomatic infection	Ref.
Immunoassay-based strips	Urine	PfHRP2	*Para*SightR^R^-F dipstick lateral flow	81%/26%/NP^a^	∼15 min (kit standard), NP	Medium	Children (*n* = 112)	East Sepik Province, Papua New Guinea	No	30
	Urine	PfHRP2	Urine malaria test (UMT) dipstick	83.75%/83.48%/NP at lower limit of detection (LLD) ∼120 parasites per μL, sensitivity 89.7% (≥201 μL^−1^)	20 min	High	Febrile patient (*n* = 195; 90.3% children < 18 years, median age 3 years, range 6 months–60 years)	Southeast Nigeria	No	37
	Urine	PfHRP2, pLDH	QDx Malaria PAN/*Pf* rapid card test (Nicholas Piramal India Ltd.)	17.3%/100%/38%	∼15 min (kit standard), NP	Medium	Febrile patients	Mumbai, India (*n* = 75 smear-positive) + controls (*n* = 25)	No	40
	Urine	PfHRP2	BinaxNOW® Malaria immunoassay-based strips kit (Inverness Medical, Europe)	86.67%/94.12%/∼90%	∼15 min (kit standard), NP	High	Febrile patients >5 years old (*n* = 60)	Rourkela, Sundargarh District, Odisha, India	No	42
	Urine	PfHRP2	Fyodor Biotechnologies UMT dipstick	79%/89%/∼90%	∼25 min (kit standard), NP	High	*n* = 1,691; ≥2 years old, 566 (34%) febrile, and 1125 (66%) afebrile	Lagos State, southwest Nigeria	Yes, detected in afebrile group (sensitivity: 72%, specificity: 91%), but lower sensitivity *vs.* febrile cases	43
	Urine	PfHRP2	CareStart™ Malaria PfHRP2 (AccessBio, USA) and Global Devices Malaria *P. falciparum*/*P. vivax* (Global Devices, USA)	Overall: 71%/96%/76%; CareStart™: 67.1%/95.2%/73.6%; Global Devices: 80%/100%/82.4%	10–20 min (kit standard)	Medium	Malaria patients (*n* = 100), negative controls (*n* = 25); ages 4 months–32 years; 76% aged 5–15 years	Ibadan, Nigeria	No	47
	Urine	PfHRP2	Fyodor® UMT (Fyodor Biotechnologies, USA/Nigeria) and SD BIOLINE Malaria Ag *Pf*/Pan (Access Bio, USA)	UMT: 55.4%/47.5%/NP; RDT: 25.4%/86.3%/NP	15–20 min (kit standard)	Medium	Adults ≥18 years with febrile illness (*n* = 384)	Ilesa, Osun State, Nigeria	No	46
	Urine	PfHRP2	Fyodor Biotechnologies UMT dipstick	85.0%/76.2%/81.9%	∼25 min (kit standard)	High	Pregnant women with clinical features of malaria (*n* = 310)	Abakaliki, Ebonyi State, Nigeria	No	49
	Saliva	pLDH	OptiMAL-IT immunoassay-based dipstick (DiaMed AG, Switzerland)	77.9%/100%/NP	∼20 min (kit standard)	Medium	Children 8 months–13 years (*n* = 130)	Ibadan, Oyo State, Nigeria	No	53
	Saliva	PSSP17	Prototype lateral flow immunoassay, mAb 27C9.B5-EuChelate particle conjugate	Asymptomatic (Cameroon/Zambia, liquid chromatography-multiple reaction monitoring (LC-MRM) ref): 91–100%/NP/NP; symptomatic (Sierra Leone, PCR ref): 83%/NP/NP	∼15 min (prototype)	High	*n* = 399; asymptomatic children (*n* = 364, 5–16 years, Cameroon & Zambia), febrile patients (*n* = 34, 3–67 years, Sierra Leone)	Cameroon (Mfou, schools), Zambia (Nchelenge District, households), Sierra Leone (Bo, Mercy Hospital clinic)	Yes, detected in 66–85% of asymptomatic carriers; limit of detection (LOD) ∼0.7 gametocytes per μL	61
	Saliva	PfHRP2	Malaria Antigen enzyme-linked immunosorbent assay (ELISA) kit (CELISA, Cellabs, Australia)	43%/100%/NP	∼2 h (ELISA protocol), NP	Low	Thick-film positive (*n* = 30) & negative children (*n* = 10) for 22 months–16 years	Korle-Bu Teaching Hospital, Accra, Ghana	No	52
	Urine, saliva	PfHRP2, pLDH	Malaria RDT kit (SD Bioline Malaria, South Korea)	Urine: 35.2%/100%/65.0%; saliva: 57.0%/100%/76.8%	∼15 min (kit standard)	High	*n* = 706 suspected malaria patients, ≥5 years	Ga West Municipality, Ghana	No	44
	Urine, saliva	PfHRP2, pLDH	Malaria immunoassay-based strip kit (SD Bioline Malaria, South Korea)	Urine: 70.7%/81.8%/85.1%; saliva: 74.5%/93.1%/88.3%	∼15 min (kit standard)	Medium	Febrile patients (*n* = 301, ages 1–92)	Buea and Tiko health districts, Southwestern Cameroon	Yes, detected 14.4% (urine malaria immunoassay-based strips) and 10.9% (saliva strips) of microscopy/immunoassay-based strip -negative but PCR-positive participants	45
Nucleic acid amplification methods	Urine, saliva	*P. falciparum* DNA (merozoite surface protein 2 (MSP2), dihydrofolate reductase (DHFR) codon 59)	Nested polymerase chain reaction (nPCR); DNA extraction *via* Qiagen DNeasy® kit or Chelex method	Qiagen kit 2.3–2.6× higher yield than Chelex; saliva yield 1.6× > urine; some microscopy-negative cases PCR-positive	∼1–2 d	Low	Febrile patients (*n* = 51, ages 1–54 years)	Southern Province, Zambia	Yes, most cases asymptomatic	31
	Saliva, urine	*P. falciparum* deoxyribonucleic acid (DNA) (18S ribosomal ribonucleic acid (rRNA) gene)	DNA extraction *via* QIAamp DNA Mini kit; nPCR qualitative; quantitative PCR (qPCR) (TaqMan, SYBR Green) quantitation	Urine: 32%/98% (poor correlation *r* = 0.20); saliva: 73%/97% (sensitivity 82% at ≥1000 parasites per μL, correlation *r* = 0.58)	∼1–2 d	Low	Febrile patients (*n* = 386, ages ≥10 years)	Gambia	Yes, detected some infections negative by blood microscopy and PCR	32
	Urine, saliva	*P. falciparum* and *P. vivax* DNA (small subunit rRNA gene)	nPCR; DNA extraction *via* Qiagen DNA Mini kit (Qiagen, Hilden, Germany)	Urine (all): *Pf* 36.2%/100%/63.3%, *Pv* 27.0%/100%/61.7%; saliva (all): *Pf* 65.2%/100%/80.0%, *Pv* 76.2%/100%/87.5%	∼1–2 d	Low	Febrile patients (*n* = 120, ages 4–60 years)	Ta Song Yang District, Tak Province, Thailand	NP	33
	Urine, saliva, buccal mucosa	*P. falciparum* DNA (merozoite surface protein 1 (MSP1), MSP2 genes)	nPCR; DNA extraction *via* Qiagen DNA Mini kit (Qiagen, Hilden, Germany)	MSP1/MSP2 gene-specific primers detected 60/63 alternative samples, independent of age, temperature, or parasite density	∼1–2 d	Low	Febrile patients (*n* = 93, ages 4–75 years)	Elkabashi Village Hospital, 80 km north of Khartoum North, Khartoum State, central Sudan	NP	34
	Urine, saliva	*Plasmodium* mitochondrial cytochrome b (*cytb*) gene	nPCR; DNA extraction *via* Qiagen DNA Mini kit (Qiagen, Hilden, Germany)	Urine: *Pf* 55.1%/100%/NP, *Pv* 53.3%/97.5%/NP; saliva: *Pf* 74.2%/100%/NP, *Pv* 79.2%/98.7%/NP; combined urine/saliva: *Pf* 85.4%/100%/NP, *Pv* 84.4%/97.5%/NP	∼1–2 d	Low	Febrile patients (*n* = 157 matched samples from Tak Province; total study *n* = 693, ages 3–78 years)	Tak, Chantaburi, Yala, and Narathiwat Provinces, Thailand	Yes, detected submicroscopic and mixed-species infections missed by microscopy	35
	Urine, saliva, buccal mucosa	*P. falciparum* Pfs16-mRNA (all gametocyte stages), *P. falciparum* surface protein 25 (Pfs25)-messenger RNA (mRNA) (mature gametocytes), 18S-rRNA (all parasite stages)	Quantitative nucleic acid sequence-based amplification (QT-NASBA); RNA extraction from filter paper using NucliSens easyMAG (bio Merieux, Lyon, France); amplification with NucliSENS EasyQ®	18S-rRNA: urine 66.7%/NP/NP; saliva 80%/NP/NP; mucosa (swab) 13.3%/NP/NP, Pfs16-mRNA: urine 20%/NP/NP; saliva 13.3%/NP/NP; mucosa (swab) 6.7%/NP/NP, Pfs25-mRNA: not detected in noninvasive samples	∼1–2 d	Moderate	Symptomatic uncomplicated *P. falciparum* patients (*n* = 15, ages 14–70 years)	Jimma Zone in Oromiya regional state, Ethiopia	NP	36
			Basic kit (bio Merieux, Lyon, France)							
	Saliva	*P. falciparum*-specific repetitive sequence 364 (*Pf*r364) chromosomal repetitive sequence (CRS); *P. vivax*-specific repetitive sequence 47 (*Pv*r47) chromosomal repetitive sequence (CRS); 18S rRNA gene	nPCR; DNA extraction *via* QIAamp DNA Isolation kit (Qiagen, Hilden, Germany); 18S nPCR; singleplex and multiplex CRS PCR	18S n-PCR: *Pv* 86.36%/98.46%/93.57%, *Pf* 100%/100%/100%; singleplex CRS: *Pf* 100%/100%/100%, *Pv* 79.55%/98.46%/90.85%; multiplex CRS: *Pf* 71.43%/100%/99.08%, *Pv* 70.45%/99.23%/87.61%	∼1–2 d	Low	Febrile individuals suspected of malaria (*n* = 327; ages 7–80); matched blood–saliva samples from *n* = 223	Delhi, Raipur, India	Yes, detected two submicroscopic *P. vivax* infections missed by microscopy	56
	Urine, saliva	*P. falciparum*, *P. vivax* DNA (18S rRNA gene)	nPCR targeting *Plasmodium*-specific outer primers (P1, P2) and species-specific primers; loop-mediated isothermal amplification (LAMP) targeting species-specific 18S rRNA	nPCR – urine: 71.0%/100%/NP; saliva: 89.4%/97.3%/NP, LAMP – urine: 29.0%/100%/NP; saliva: 47.0%/100%/NP	∼1–2 d (nPCR); ∼2 h (LAMP)	PCR: low, LAMP: moderate	Febrile patients (*n* = 108; ages 3–57 years)	Sistan and Baluchestan Province, southeastern Iran	NP	38
	Urine, saliva	*P. falciparum*, 2*P. vivax* DNA (mitochondrial *cytb* gene)	DNA extraction *via* QIAamp DNA Mini kit (Qiagen, Hilden, Germany) for blood/saliva; QIAamp Viral RNA kit (Qiagen, Hilden, Germany) for urine	Urine: 70%/97%/NP; saliva: 91%/97%/NP	∼1–2 d	Low	Febrile patients (*n* = 99)	Chabahar and Sarbaz Districts, Sistan and Baluchestan Province, Iran	Yes, detected submicroscopic and mixed-species infections missed by microscopy	39
	Saliva	*P. falciparum* DNA (multi-copy 18S rRNA gene)	nPCR: OMNIgene®·ORAL kit (OM-501; DNA Genotek, Ottawa, Canada); DNA extraction *via* Chelex-100; nested PCR amplification	*vs.* microscopy: 95%/93%/94%; *vs.* nPCR-blood: 82%/99%/93%	∼1–2 d	Low	Febrile patients (*n* = 222; ages 2–76 years)	Far North, Centre, and Northwest Regions, Cameroon	Yes, detected 16 submicroscopic infections missed by microscopy	57
	Saliva, urine	*P. falciparum* genomic DNA; *P. falciparum* chloroquine resistance transporter (pfcrt), *P. falciparum* dihydrofolate reductase-thymidylate synthase (pfdhfr-ts), *P. falciparum* Kelch 13 (pfK13) propeller genes	nPCR: DNA extraction from filter paper using Chelex100; nested PCR with gene-specific primers	Urine: *pfK13*: 45%/50%/45%, *pfdhfr*: 38%/50%/38%, *pfcrt*: 0%/1%/1%; saliva: *pfK13*: 46%/20%/43%, *pfdhfr*: 64%/50%/54%, *pfcrt*: 5%/50%/5%	∼1–2 d	Low	Symptomatic *P. falciparum*-positive patients (*n* = 153, ages >2 years)	Anonkoua-Kouté, Port-Bouët, and Ayamé, Côte d'Ivoire	No	41
	Saliva	*P. falciparum* mitochondrial cytochrome c oxidase subunit 3 (*cox3*) gene, nuclear *var acidic terminal sequence* (*varATS*) gene, 18S rRNA gene	nPCR: OMNIgene·ORAL kit (DNA Genotek, Canada); DNA extraction with NucleoSpin kit (Macherey-Nagel, Germany)	18S rRNA: 62%/NP/NP; cox3: 77%/NP/NP; *varATS*: 68%/NP/NP (all *vs.* blood PCR reference)	∼1–2 d	Low	Febrile patients (*n* = 60)	Cameroon	Yes, detected submicroscopic infections (up to 47% with *cox3*)	58
	Hair	*Plasmepsin* 4 gene	qPCR: UltraClean Blood Spin kit (Mo Bio, USA) DNA extraction; PrimerDesign qPCR kit (GeneSig, UK); iTaq Universal Probes Supermix (Bio-Rad, USA); StepOnePlus Real-Time PCR System (Applied Biosystems, USA)	36.84%/NP/NP	∼1 d	Low	Malaria patients (*n* = 19; ages 4–31 years)	Gakenke District, Rwanda	No	87
	Saliva	*Pv*r47 (*P. vivax*), *Pf*r364 (*P. falciparum*)	qPCR: ViiA 7 Real-Time PCR System (Thermo Fisher Scientific); QX200 Droplet Digital PCR System (Bio-Rad)	qPCR: 77%/NP/NP; droplet digital polymerase chain reaction (ddPCR): 73%/NP/NP	∼1 d	Low	Symptomatic patients (*n* = 157) and healthy controls (*n* = 30)	Rondônia & Roraima, Brazil	Yes, lower sensitivity in <1500 parasites per μL	62
Biosensors	Saliva	immunoglobulin G (IgG) antibodies to *P. falciparum* apical membrane antigen 1 (AMA-1) and merozoite surface protein 1 19 kDa fragment (MSP-1_19_)	ELISA	AMA-1: 64–77%/91–100%/NP; MSP-1_19_: 47–67%/90.0–97%/NP	∼5–6 h	High	Tanzania: adults with child <5 (*n* = 53); Gambia: children ages 1–15 years (*n* = 200)	Ihanda & Ndurugumi villages, Dodoma Region, Tanzania; Farafenni area, The Gambia	Yes, detects prior exposure, applicable in low-transmission settings	54
	Saliva	PfHRP2	Chemiluminescent ELISA (sandwich)	PfHRP2 concentration range: 17–1167 pg mL^−1^, 100%/100%/NP	∼5–6 h	Moderate	Febrile patients (*n* = 8) and healthy controls (*n* = 16)	Roxas, Palawan in Philippines; Los Angeles, USA (controls)	NP	56
	Saliva	PfHRP2	ELISA (sandwich)	88.5%/100%/94%	∼5–6 h	Moderate	Febrile patients (*n* = 96)	Kisumu County Kenya	NP	64
	Saliva	*Plasmodium* topoisomerase I enzymatic activity	Rolling Circle Enhanced Enzyme Activity Detection (REEAD) sensor	100% (35/35 positives)/100% specificity (29/29 negatives)/NP	<1 h	Moderate	Febrile patients (*n* = 35 positive, *n* = 29 negative)	Lambaréné, Gabon	NP	59
	Saliva	PfHRP2	Label-free impedimetric biosensor (interdigitated electrode, IDE)	LOD 25 pg mL^−1^ in saliva; detected PfHRP2 in 8/11 spiked clinical isolate samples	∼2 h	Moderate	Healthy adults (saliva spiked with PfHRP2 from clinical isolates and lab strains)	Papua New Guinea, Malawi	NP	60
	Saliva	*Plasmodium* topoisomerase I (pTopI) activity	DNA-based REEAD with chemiluminescence readout	90.9% (30/33)/42.9% (3/7)/NP	∼3–4 h	High	Febrile patients (*n* = 33) and presumed asymptomatic negatives (*n* = 7)	Lambaréné, Gabon	Yes, detected in presumed asymptomatic cases	63
Gas chromatography-mass spectrometry (GC-MS) based on VOCs	Exhaled alveolar breath	VOCs (thioethers: allyl methyl sulfide, 1-methylthio-propane, (*Z*)/(*E*)-1-methylthio-1-propene)	GC-MS analysis with machine-learning classification	Perfect classification (100% accuracy in cross-validation); no conventional sensitivity/specificity reported; detectable at ∼0.001% parasitemia	∼1 h	Low	Healthy malaria-naïve adults (*n* = 13, induced blood-stage malaria (IBSM) model)	Australia (clinical trial setting)	NP	71
	Exhaled breath	Six VOCs: methyl undecane, dimethyl decane, trimethyl hexane, nonanal, isoprene, tridecane; also elevated α-pinene & 3-carene (mosquito attractants)	GC-MS + correlation-based classifier analysis (nearest mean)	71%/94%/83%	∼1 h	Low	Febrile children (ages 3–15 years) with and without uncomplicated *P. falciparum* malaria (*n* = 35)	Lilongwe, Malawi	NP	72
	Skin volatiles (arm and foot)	Multiple VOCs (*e.g.*, hexanal, octanal, decane, toluene, benzaldehyde, 2-ethylhexan-1-ol)	GC-MS + genetic algorithm (GA) models	Fever group (arm): 100/100/100%; any symptom (arm): 85.7/60/75%; any symptom (foot): 57.1/80/66.7%; diarrhea group: 100/50/75% (arm)	∼1 h	Low	Febrile children (*n* = 114)	Mbita, Kenya	NP	74
	Skin volatiles (foot and arm)	Multiple skin-emitted VOCs (*e.g.*, hexanal, nonanal, toluene, ethylbenzene, 2-ethylhexan-1-ol, 4-hydroxy-4-methylpentan-2-one)	GC-MS + machine learning (Adaboost, random forest (RF))	Foot VOCs: asymptomatic malaria-infected individuals (AS) *vs.* uninfected individuals (U): 100%/NP/78 %, symptomatic malaria-infected individuals (S) *vs.* U: 91/NP/85 %, all S *vs.* U: 95/NP/77 %;arm VOCs: AS *vs.* U: 75/NP/78 %, S *vs.* U: 89/NP/89 %, all S *vs.* U: 92/NP/80 %	∼1 h	Low	Febrile (ages ≤12 years), *n* = 330 for main analysis + 66 submicroscopic cases (PCR+/microscopy−)	Mbita, Kenya	Yes, detected asymptomatic and submicroscopic infections with 100 % sensitivity (foot samples)	75

a NP: not provided.

#### 3.1.1. Immunoassay-based strips

**Fig. 2:**
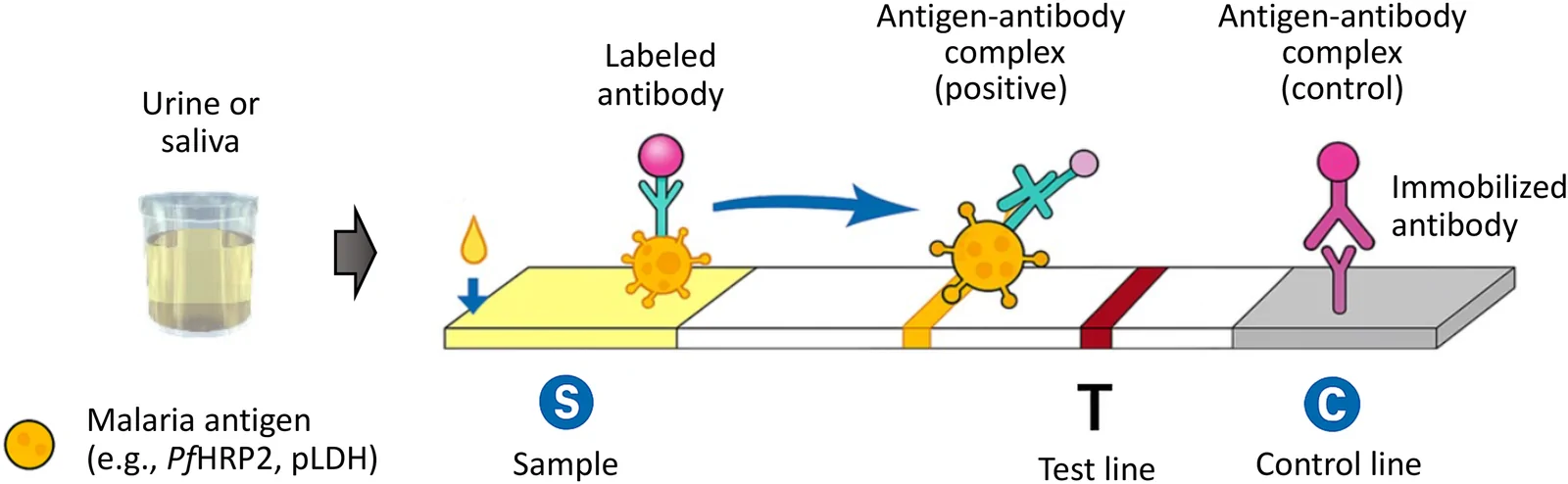
Immunoassay-based strips for noninvasive malaria detection. Schematic illustration of a lateral flow immunoassay-based strip using noninvasive samples (*e.g.*, urine or saliva). When the sample is applied to the sample pad S, *Plasmodium*-derived antigens (*e.g.*, *P. falciparum* histidine-rich protein 2 (PfHRP2) or parasite lactate dehydrogenase (pLDH)), if present, bind to labeled detection antibodies. The resulting antigen–antibody complex migrates along the nitrocellulose membrane *via* capillary action. At the test line T, the complex is captured by immobilized secondary antibodies, generating a visible signal that indicates a positive result. The control line C captures unbound labeled antibodies to confirm proper test function. Original illustration created by the authors in part using https://BioRender.com.

Immunoassay-based strips enable fast and simple detection of malaria infections by identifying soluble parasite-derived antigens.^2,112,113^ Initially developed for blood,^112,113^ immunoassay-based strips have also been adapted to noninvasive fluids (*e.g.*, urine and saliva) by targeting *P. falciparum* histidine-rich protein 2 (PfHRP2) and/or parasite lactate dehydrogenase (pLDH), which can be detected in these fluids during active infection. Detection strips function through a lateral flow immunoassay mechanism (Fig. 2). When a noninvasive sample (*e.g.*, urine or saliva) is applied to the sample pad (S), any malaria antigens present (*e.g.*, PfHRP2 or pLDH) bind to labeled detection antibodies (*e.g.*, conjugated to nanoparticles). This antigen–antibody complex migrates along the nitrocellulose membrane by capillary action. At the test line (T), the complex is captured by a second, immobilized antibody, producing a visible colored line that indicates a positive result. A separate control line (C) captures excess labeled antibodies to verify that the test has run correctly.

Multiple studies have evaluated the feasibility of applying immunoassay-based strips to urine and saliva samples. Urine strips are the most extensively studied with *Para*Sight^R^-F,^30^ urine malaria test (UMT),^37,43,46,49^ and QDx malaria PAN *P. falciparum* (PAN/*Pf*)^40^ tested in endemic regions including Nigeria, India, and Papua New Guinea. These assays typically target soluble parasite antigens, which can be detected in urine during infection. Saliva strips detect the same core biomarkers (*i.e.*, PfHRP2, pLDH)^52,53^ as well as gametocyte-associated proteins, PSSP17,^61^ which has been evaluated for identifying infectious and subclinical cases. Dual-sample strategies combining urine and saliva were introduced to improve detection yield, but sensitivity gains over single-sample testing were marginal and often limited by antigen concentration and sample variability.^44,45^

The detection performance of urine strips has varied widely across studies. Genton *et al.*^30^ first applied the *Para*Sight^R^-F test in Papua New Guinea, reporting 81% sensitivity but only 26% specificity. In Nigeria, Oguonu *et al.*^37^ found the urine-based UMT had 83.8% sensitivity and 83.5% specificity, with a detection limit of ≃120 parasites per μL and sensitivity increasing at higher densities. In a larger trial, Oyibo *et al.*^43^ reported 79%/89% overall and 85%/84% in febrile patients, comparable to blood RDTs; accuracy declined in afebrile cases, but remained unchanged after PCR correction. Samal *et al.*^42^ in India observed 86.7% sensitivity and 94.1% specificity using BinaxNOW, while Abiodun *et al.*^47^ in Nigeria found 71% sensitivity and 96% specificity. By contrast, Anchinmane *et al.*^40^ showed that the QDx malaria PAN/*Pf* test achieved only 17.3% sensitivity despite 100% specificity. More recently, Oyeniyi *et al.*^46^ found low agreement with microscopy: the urine UMT showed 55.4% sensitivity and 47.5% specificity (Cohen's kappa *κ* = 0.03), while the blood RDT showed 25.4% sensitivity and 86.3% specificity (*κ* = 0.10). Given that Cohen's kappa quantifies concordance beyond chance, these low coefficients indicate that both tests exhibit only minimal true concordance with microscopy. Overall, urine strips' performance is heterogeneous and strongly dependent on parasite density (≤200 μL^−1^ often poor), target chemistry (PfHRP2 *vs.* pLDH), and study context. Recently, Ozonu *et al.* in Nigeria reported that UMT showed 85.0% sensitivity and 76.2% specificity with overall accuracy comparable to blood-based RDT (81.9% *vs.* 84.8%; area under the curve 0.83 *vs.* 0.86).^49^

As an early proof-of-concept, Wilson *et al.*^52^ used a custom chemiluminescent enzyme-linked immunosorbent assay (ELISA) to detect PfHRP2 in saliva, reporting 43% sensitivity and 100% specificity. Building on this finding, saliva strips demonstrated substantial variability in sensitivity, ranging from 7.9% to over 90%, depending on the sample type and targeted biomarker.^53,61^ Gbotosho *et al.*^53^ evaluated the OptiMAL-IT dipstick for pLDH detection and found only 7.9% sensitivity with spun saliva, which increased to 77.9% when whole saliva was used, highlighting the importance of sample processing. By contrast, Tao *et al.*^61^ developed a lateral flow assay targeting the gametocyte-specific marker PSSP17 and achieved sensitivities above 90%, providing field evidence for detecting infectious and asymptomatic carriers. To enhance detection yield, Aninagyei *et al.*^44^ in Ghana evaluated urine and saliva using modified immunoassay-based strip protocols and reported modest sensitivities (35.2% for urine, 57.0% for saliva), with performance largely dependent on high parasitaemia, anemia, or occult blood contamination. Similarly, Apinjoh *et al.*^45^ in Cameroon applied standard PfHRP2/pLDH-based strips to both fluids and observed improved but still suboptimal results (70.7% sensitivity for urine, 74.5% for saliva compared to PCR), underscoring the need for optimized nonblood assay designs.

Noninvasive immunoassay-based strips retain practical advantages for field deployment but face several limitations. Sensitivity drops significantly at low parasite densities (≤200 parasites per μL), sometimes below 20%, largely due to low antigen concentrations in urine and saliva.^37,42–44,47^ Additional factors including protein degradation, salivary contamination, and individual variability can reduce detection consistency.^47,53,61^ Variability may also arise from circadian fluctuations, hydration-dependent dilution, and parasite sequestration, while high temperature and humidity can further compromise antigen stability. In general, *pfhrp2/pfhrp3* gene deletions in parts of sub-Saharan Africa undermine the accuracy of HRP2-based tests, and HRP2 antigens may persist post-treatment, complicating monitoring.^112,113^ These limitations collectively constrain the sensitivity and reliability of noninvasive immunoassay-based strips, particularly in low-density and asymptomatic infections.^2,3^

Despite these challenges, immunoassay-based strips remain attractive due to their ease of use, lack of infrastructure requirements, and short turnaround time (15–20 minutes).^112,113^ Their high specificity (83–96%) and noninvasive format support repeat testing and broad acceptability, making them particularly suitable for pediatric and remote populations.^25,37,42,43^ However, equitable access may still be limited by infrastructure, supply chains, community-level factors, and sex-related barriers. Immunoassay-based strips may also serve as valuable complements to blood-based detection, especially for decentralized surveillance, large-scale screening, and transmission-blocking strategies where validated.^61^ Immunoassay-based strips are primarily aligned with case management and control, given their rapid, low-cost, and field-deployable nature. However, their utility in elimination-stage settings can be limited by reduced sensitivity at low parasite density.

#### 3.1.2. Nucleic acid amplification methods

**Fig. 3:**
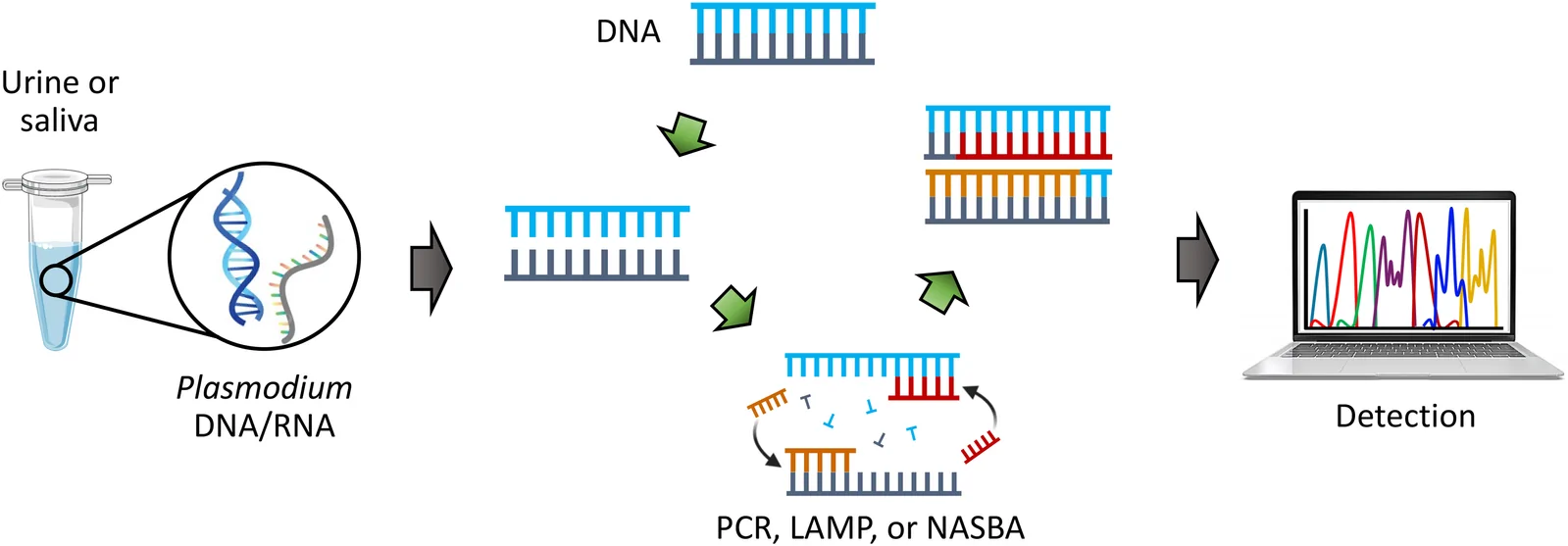
Nucleic acid amplification methods for noninvasive malaria detection. *Plasmodium* deoxyribonucleic acid (DNA) or ribonucleic acid (RNA) can be extracted from nonblood samples (*e.g.*, urine or saliva) and amplified using primers targeting conserved genomic regions. Polymerase chain reaction (PCR) relies on repeated thermal cycling (denaturation, annealing, and extension), whereas loop-mediated isothermal amplification (LAMP) and nucleic acid sequence-based amplification (NASBA) are performed under isothermal conditions. NASBA specifically amplifies RNA transcripts *via* reverse transcription, ribonuclease H (RNase H) digestion, and T7 RNA polymerase-driven replication. The resulting products can be detected using fluorescence readouts, gel electrophoresis, or sequencing analysis. Original illustration created by the authors in part using https://BioRender.com.

Nucleic acid amplification methods (*e.g.*, PCR, LAMP, and NASBA) are powerful tools for detecting malaria.^19,20,114^ These methods amplify parasite-derived deoxyribonucleic acid (DNA) or ribonucleic acid (RNA) by targeting conserved genomic regions with specific primers (Fig. 3), enabling detection even at very low parasite densities. PCR relies on thermal cycling with denaturation, annealing, and extension, whereas LAMP amplifies DNA at a constant temperature, allowing faster and simpler workflows. NASBA also functions isothermally but targets RNA using reverse transcriptase, ribonuclease H (RNase H), and T7 RNA polymerase, making it particularly suitable for detecting active transcripts (*e.g.*, gametocyte-specific messenger RNA (mRNA)). Detection of amplified products may involve gel electrophoresis, fluorescence measurement, or sequencing analysis. Because the evidence base is uneven, PCR, LAMP, and NASBA should not be treated as equally validated in nonblood malaria samples; the strongest clinical evidence remains PCR-based, while LAMP and NASBA currently have fewer nonblood studies and more variable performance.

Several studies have employed nucleic acid amplification on saliva samples with encouraging detection performance. Najafabadi *et al.* directly compared nested PCR and LAMP in matched saliva and urine samples; nested PCR outperformed LAMP, whereas LAMP retained high specificity but lower sensitivity, particularly in urine, illustrating the trade-off between simplified isothermal amplification and analytical sensitivity in nonblood matrices.^38^ Nwakanma *et al.*^32^ similarly showed that saliva-based nested PCR achieved 73% sensitivity and 97% specificity, despite parasite DNA being over 600-fold lower than in blood. Singh *et al.*^56^ found that nested 18S PCR in saliva achieved the highest sensitivity (87.4%) compared to singleplex and multiplex formats. Najafabadi *et al.*^39^ further demonstrated that mitochondrial nested PCR achieved 91% sensitivity in saliva and 70% in urine, both with 97% specificity. Mfuh *et al.*^57^ reported that saliva-based nested PCR maintained 96.8% sensitivity and 100% specificity even after room-temperature storage, highlighting its field applicability. Lloyd *et al.*^58^ applied ultrasensitive PCR targeting mitochondrial *cytochrome c oxidase subunit 3* (*cox3*) and *var acidic terminal sequence* (*varATS*), detecting *Plasmodium* DNA in 77% and 68% of saliva samples, respectively. More recently, Costa *et al.*^62^ used droplet digital PCR (ddPCR) and quantitative PCR (qPCR) on saliva and buccal swabs using multicopy repetitive sequences (*P. vivax* repetitive sequence 47 (*Pvr47*), *P. falciparum* repetitive sequence (*Pfr364*)), achieving high sensitivity at low parasite densities and underscoring the quantitative precision of droplet digital PCR (ddPCR).

Studies using urine and multi-sample approaches have shown more variable outcomes. Mharakurwa *et al.*^31^ amplified *P. falciparum* merozoite surface protein 2 (MSP2) and dihydrofolate reductase (DHFR) from urine and saliva by nested PCR, but detection was inconsistent in low parasitemia and declined with longer amplicons. Buppan *et al.*^33^ detected *P. falciparum* and *P. vivax* DNA in both fluids, with higher sensitivity in ethanol-preserved saliva (84%) compared to urine (34%). A-Elgayoum *et al.*^34^ applied *P. falciparum* merozoite surface protein 1 (MSP1)/MSP2 nested PCR to saliva, urine, and buccal mucosa, detecting *P. falciparum* DNA in 60 of 63 samples, while species-specific primers performed poorly in nonblood matrices. Putaporntip *et al.*^35^ reported that *cytochrome b* (*cytb*)-based nested PCR detected malaria in 74% of saliva and 53% of urine samples, outperforming 18S PCR and microscopy at low parasitemia. Dagnogo *et al.*^41^ achieved amplification in 40% of saliva *versus* 27% of urine samples, underscoring sample-type effects on DNA recovery. Similarly, Nwakanma *et al.*^32^ observed poor urine sensitivity (32%), reflecting parasite DNA concentrations ∼2500-fold lower than in blood.

Less conventional nonblood matrices have also been investigated. Kast *et al.*^36^ applied quantitative nucleic acid sequence-based amplification (QT-NASBA) to detect gametocyte transcripts but found very low sensitivity in nonblood samples, with *P. falciparum* vacuole membrane protein (Pfs16) mRNA detected in only 13% of saliva and 20% of urine samples, while *P. falciparum* surface protein 25 (Pfs25) mRNA was undetectable. Thus, NASBA is conceptually attractive for RNA-based transmission markers, but the available nonblood data suggest that RNA degradation and low transcript abundance remain major barriers. Gómez-Luque *et al.*^87^ tested hair as a noninvasive sample using qPCR and reported only moderate sensitivity (36.84%), likely reflecting low parasite DNA abundance in hair follicles. Overall, PCR generally provides superior sensitivity compared to LAMP or NASBA, especially when multicopy targets (*e.g.*, 18S rRNA, mitochondrial genes) are used. Optimized sample preparation methods, including dried saliva spots, whole-mouth saliva supernatant, and silica-based purification, can further enhance detection yield.^32,35,57,58^ Importantly, studies by Putaporntip,^35^ Kast,^36^ Mfuh,^57^ and Costa^62^ demonstrated that nonblood PCR can detect infections in asymptomatic carriers, supporting its proposed use for early detection and community-level screening.

Nevertheless, key operational challenges remain. Saliva, urine, and buccal samples typically contain lower parasite nucleic acid concentrations than blood, and quality is influenced by hydration, oral health, and collection timing.^32,35^ RNA-based methods (*e.g.*, NASBA) are particularly vulnerable to degradation and often require cold storage, limiting field feasibility.^36^ Moreover, many platforms depend on thermal cyclers or fluorescence readers and require trained personnel and stable power supplies. Ongoing innovations, including real-time LAMP, dried sample formats, and portable PCR devices, aim to overcome these limitations. As emphasized in prior reviews,^62,114^ improving assay robustness and field adaptability will be crucial for enabling accurate, noninvasive malaria testing.

#### 3.1.3. Biosensors

Biosensors are compact analytical devices that use biologically derived recognition elements (*e.g.*, antibodies aptamers, enzymes, and nucleic acids) to detect specific analytes and convert this interaction into measurable signals through electrochemical, optical, or mechanical means.^115–118^ In noninvasive malaria detection, biosensors adapted to saliva or urine offer a practical alternative to conventional blood-based assays.^54,55,59,60,63^

**Fig. 4:**
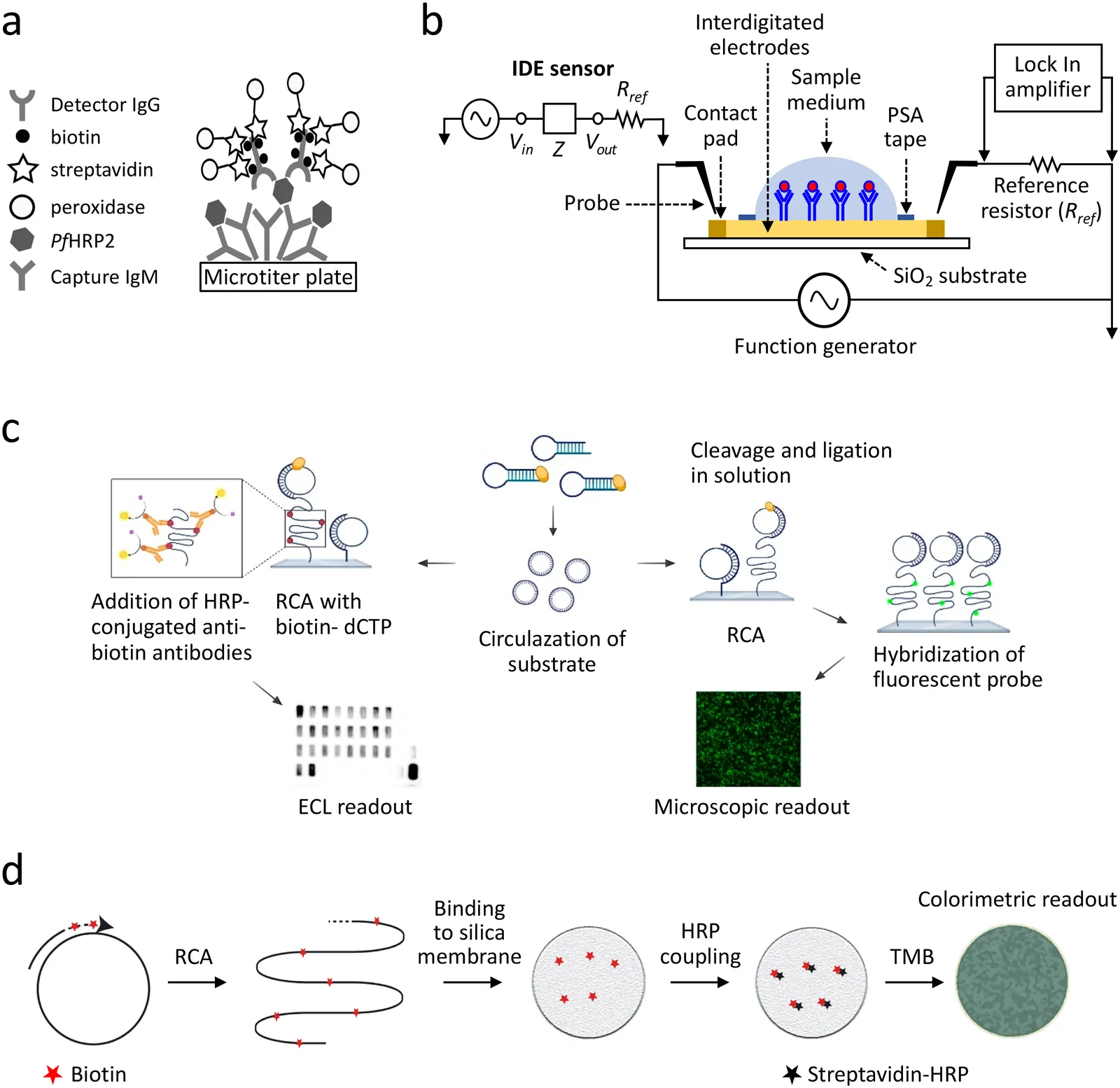
Representative biosensors for noninvasive malaria detection. (a) Enzyme-linked immunosorbent assay (ELISA)-based biosensor.^55^ Sandwich ELISA configuration for detecting *P. falciparum* histidine-rich protein 2 (PfHRP2), in which immobilized immunoglobulin M (IgM) capture antibodies (Ab) bind the target antigen and biotinylated immunoglobulin G (IgG) detector antibodies recognize a separate epitope. Signal amplification is achieved *via* a streptavidin-peroxidase complex, enabling multivalent epitope recognition [adapted from ref. 55 under the terms of the CC BY 2.0 license; © 2012 The Author(s).]. (b) Interdigitated electrode (IDE)-based biosensor.^60^ Impedance biosensor using IDE to measure resistance changes upon PfHRP2 binding. The system includes a function generator, reference resistor, and lock-in amplifier, with sensing performed through a sample chamber defined by pressure sensitive adhesive (PSA) tape [adapted from ref. 60 under the terms of the CC BY 4.0 license; © 2019 The Author(s).]. (c) Rolling-circle enhanced enzyme activity detection (REEAD)-based biosensor.^63^ REEAD system detects *Plasmodium* topoisomerase I (pTOP1) activity in saliva by converting a deoxyribonucleic acid (DNA) substrate into a circular form *via* cleavage-ligation, followed by rolling circle amplification (RCA). Signal is visualized through either electrochemiluminescence (ECL) or fluorescence microscopy [Adapted from ref. 63 under the terms of the CC BY 4.0 license; © 2024 The Author(s).]. (d) Colorimetric REEAD-based biosensor.^59^ pTOP1 generates circular DNA templates that undergo rolling circle amplification with biotinylated nucleotides. The amplified products are immobilized on a silica membrane, coupled with streptavidin-HRP, and visualized by 3,3′,5,5′-tetramethylbenzidine (TMB), producing a naked-eye colorimetric signal [adapted from ref. 59 under the terms of the CC BY 4.0 license; © 2018 The Author(s).].

Biosensor systems have been developed to target various malaria-associated biomarkers (*e.g.*, PfHRP2), parasite lactate dehydrogenase, and *Plasmodium*-specific nucleic acids or enzymes (Fig. 4). In the sandwich-type enzyme-linked immunosorbent assay (ELISA) system reported by Fung *et al.*,^55^ PfHRP2 in saliva is captured by immobilized immunoglobulin M (IgM) and detected by a biotinylated immunoglobulin G (IgG) coupled with streptavidin-linked peroxidase, enabling multivalent recognition and signal amplification (Fig. 4a). The assay detected PfHRP2 within a range of 17–1167 pg mL^−1^, achieving 100% sensitivity and specificity. Estévez *et al.*^54^ evaluated a saliva-based ELISA targeting *P. falciparum* apical membrane antigen 1 (AMA-1) and merozoite surface protein 1 19-kDa fragment (MSP-1_19_) (sensitivity ranges 64–77% and 47–67% and specificity ranges 91–100% and 90–97%, respectively), with strong correlation between saliva and plasma antibody levels. Bittok *et al.*^64^ evaluated a saliva-based sandwich ELISA targeting PfHRP2 in febrile patients, reporting 88.5% sensitivity and 100% specificity with an overall diagnostic accuracy of 94% and no significant difference compared to microscopy (*p* > 0.05).

Optical and electrochemical biosensors have expanded noninvasive malaria detection by enabling sensitive and portable detection mechanisms. Soraya *et al.*^60^ developed an impedimetric biosensor using interdigitated electrode (IDE) to monitor PfHRP2 binding as resistance changes, achieving a 25 pg mL^−1^ detection limit in saliva and identifying 8 of 11 spiked clinical samples (Fig. 4b). Molecular approaches based on *Plasmodium* topoisomerase I (pTopI) activity have also produced encouraging early performance. Juul-Kristensen *et al.*^63^ applied a rolling-circle enhanced enzyme activity detection (REEAD) system with chemiluminescent readout and a portable device, clearly distinguishing positive from negative saliva samples, showing *p* < 0.0001 (Fig. 4c). Similarly, Hede *et al.*^59^ demonstrated a REEAD assay with colorimetric visualization *via* streptavidin-horseradish peroxidase (HRP)/3,3′,5,5′-tetramethylbenzidine (TMB), enabling naked-eye readout and 100% sensitivity in saliva from 35 infected individuals (Fig. 4d).

Biosensors for noninvasive malaria detection face notable technical and operational challenges. ELISA-based platforms, although specific, require multiple incubation steps and cold-chain reagents, which limit their field applicability.^54,55^ Molecular platforms (*e.g.*, REEAD) offer high specificity but involve complex amplification workflows and are sensitive to storage and enzyme stability, which complicates their deployment in low-resource settings.^59,63^ Electrochemical sensors are portable and label-free, yet remain vulnerable to electrode fouling, matrix interference, and signal drift in complex saliva samples.^60^ Notably, most studies have been conducted under controlled laboratory conditions or with spiked samples, and only a few platforms have demonstrated validation in clinical saliva specimens, underscoring the need for field-ready systems evaluated in asymptomatic or pediatric populations.^55,59,63^ To address these challenges, recent advances emphasize the integration of microfluidics, lyophilized reagents, and portable readout devices to enhance robustness and scalability for decentralized surveillance and community-based screening.^115,116^

#### 3.1.4. Gas chromatography-mass spectrometry (GC-MS) using volatile organic compounds

**Fig. 5:**
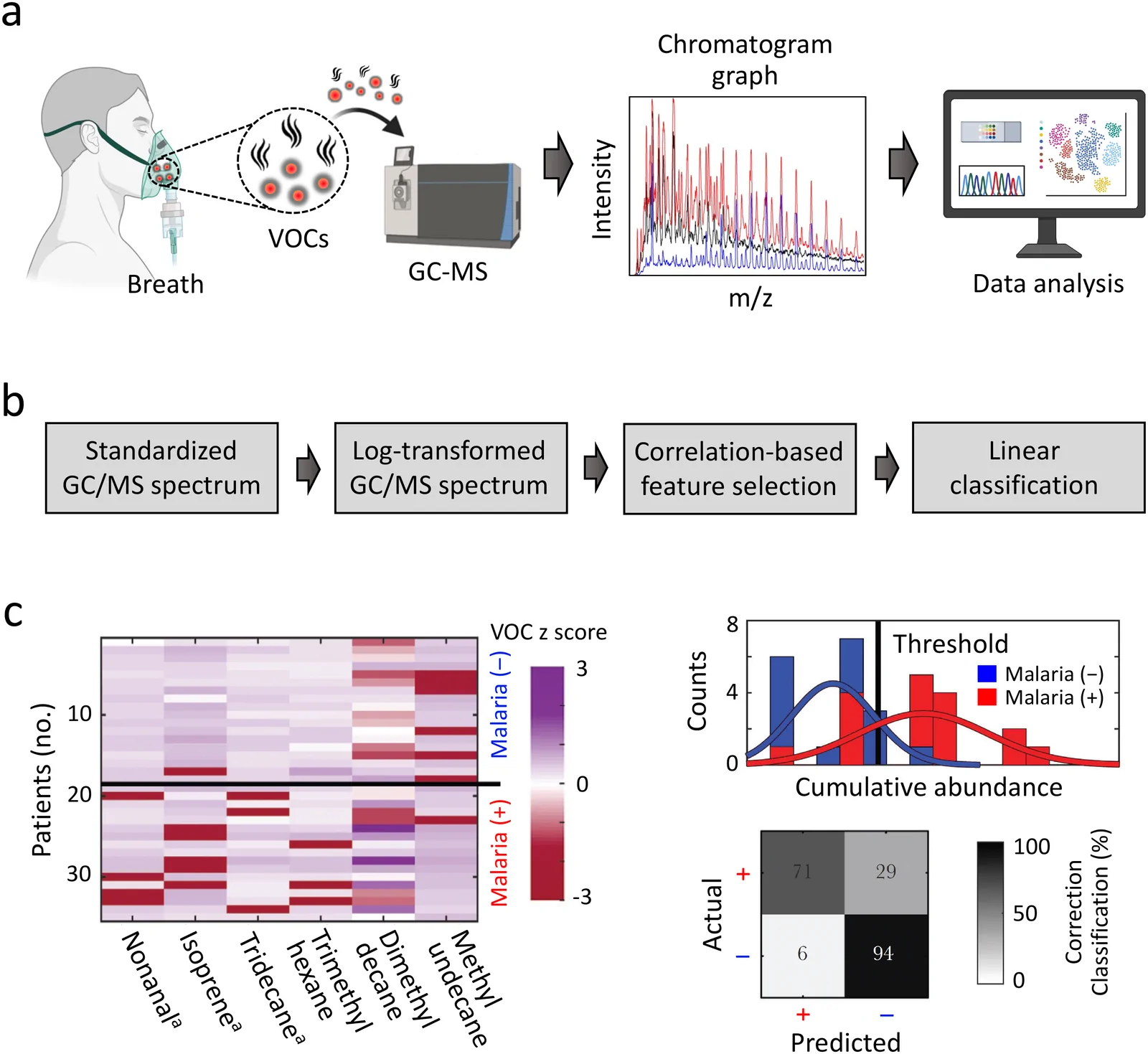
Gas chromatography-mass spectrometry (GC-MS)-based detection using malaria-associated volatile organic compounds (VOCs). (a) Schematic illustration of the GC-MS workflow for identifying malaria-related VOCs emitted from exhaled breath or skin odor. VOCs are captured onto sorbent materials, separated by gas chromatography, and analyzed *via* mass spectrometry to generate compound-specific chromatograms. Data are then processed to extract and classify biomarker patterns. Original illustration created by the authors in part using https://BioRender.com. (b and c) Breath VOC-based malaria detection.^72^ (b) Data analysis pipeline showing standardized spectral transformation, correlation-based biomarker selection, and linear classification. (c) Left: Heatmap of *z*-score normalized abundance of six discriminant VOCs across malaria-positive (red) and -negative (blue) subjects. ^a^ Compound identity confirmed by comparison to pure standard. Right: Histogram showing separation of subjects based on cumulative biomarker abundance and optimal classification threshold (top) and confusion matrix of predicted *vs.* actual infection status showing 94% specificity and 83% overall accuracy (bottom) [adapted from ref. 72 with permission from Oxford University Press; © 2018 Oxford University Press].

Gas chromatography-mass spectrometry (GC-MS) is the gold standard analytical technique for detecting and quantifying volatile organic compounds (VOCs) in complex biological samples.^68–70^ The method combines the physical separation capability of GC with the molecular specificity of MS, allowing sensitive and selective detection of minute chemical constituents. In malaria detection, GC-MS enables characterization of VOC profiles emitted in breath or skin that reflect infection-associated metabolic changes. As illustrated in Fig. 5a, VOCs are collected on sorbent tubes or solid-phase microextraction (SPME) fibers, thermally desorbed, separated by the GC column, and then ionized and fragmented in the MS for *m*/*z* analysis. This generates compound-specific spectra that can be matched to reference libraries for metabolite identification. With high sensitivity and chemical specificity,^68^ GC-MS serves as a powerful tool for biomarker discovery and disease profiling. Although VOCs have been widely explored in other disease contexts, their applications to malaria have become increasingly established through accumulating evidence from both experimental and clinical research.^71,72,74,75^

Early clinical and experimental studies demonstrated that breath-based VOCs could serve as detection indicators of malaria infection. Schaber *et al.*^72^ conducted a clinical study in Malawi, where log-transformed GC-MS spectra from children were analyzed using correlation-based feature selection and linear classification (Fig. 5b). A six-compound breath panel, summarized in Fig. 5 and Table 3, showed distinct *z*-scored abundance patterns between malaria-positive and -negative groups (Fig. 5c). A cumulative abundance metric derived from these features enabled classification with 83% accuracy, 94% specificity, and 71% sensitivity. Building on these findings, a controlled human infection study by Berna *et al.*^71^ identified four novel thioether VOCs in the breath of experimentally infected individuals. Their levels followed a 48-hour cycle aligned with parasitemia and showed strong correlation with parasite dynamics, suggesting an *in vivo* host-parasite metabolic origin.

**Table 3:** Summary of studies on acoustic, photoacoustic, and optical systems for noninvasive malaria detection

Detection method	Sample type	Biomarker & species	Platform detail	Performance (sensitivity/specificity/accuracy)	Detection time	Low-resource field usability	Research population	Research area	Detect asymptomatic infection	Ref.
Ultrasound	Abdomen (spleen, liver)	Splenomegaly, hepatomegaly, portal vein diameter	Abdominal ultrasound (splenic)	Splenomegaly detected in 82.8% overall (100% in children); more frequent in non-immune *vs.* semi-immune patients (91.0% *vs.* 65.8%); persisted in all recrudescence/relapse cases at day 21 (100%) *vs.* 59.3% in cured patients	<30 min	Moderate – portable	Febrile patients (*n* = 118, ages 2–78 years; *n* = 13 children)	Germany (patients mostly infected, Africa)	NP^a^	76
	Eye (optic nerve), brain vasculature, heart, abdomen (spleen)	Increased optic nerve sheath diameter (ONSD) (raised intracranial pressure (ICP)), middle cerebral artery (MCA) flow velocity/PI, ventricular function, splenomegaly	ONSD, Transcranial Doppler ultrasonography (TCD), Cardiac ultrasound, Abdominal ultrasound	Increased ONSD in 25% overall and 100% in cerebral malaria; abnormal MCA velocity in 29.1%; splenomegaly 24% by US	≤12 h from presentation	High	Febrile patients (*n* = 33 children, ages 6 months–10 years)	Mulago Hospital, Kampala, Uganda	NP	78
	Eye (optic nerve)	Enlarged ONSD indicating raised ICP	ONSD	Raised ICP in 49% (ONSD ≥ 4.3 mm); neurological sequelae more frequent with enlarged ONSD	<10 min	High	Febrile patients (*n* = 112 children)	Queen Elizabeth Central Hospital, Blantyre, Malawi	NP	79
	Kidney (*in vivo* imaging)	Renal size, cortical thickness, and volume	Abdominal ultrasound (renal)	Increased renal size and cortical thickness, most marked in complicated malaria; parenchymal echogenicity in 14.1% of complicated cases	Within 24 h of hospital presentation	High	Febrile patients (*n* = 170 children)	Ibadan, Nigeria	NP	80
	Spleen	Splenic volume (cm^3^) and enlargement	Abdominal ultrasound (splenic)	Ultrasound more precise than palpation; splenic volume fell 50% by day 14 in moderate malarial anemia (MMA) but unchanged in severe malarial anemia (SMA) despite transfusion	Days 0, 1, 2, 3, 7, 14, 28, 42 (serial follow-up)	High	Febrile patients (*n* = 25 children, ages < 10 years)	Papua New Guinea	NP	82
	Brain (middle cerebral arteries, basilar artery, extracranial internal carotid arteries)	Cerebral blood flow velocity patterns & autoregulation status	TCD	Five TCD phenotypes identified in cerebral malaria; mortality highest in low flow (32%), neurologic sequelae highest in vasospasm (45%); 80% had impaired autoregulation on day 1	Initial scan within 12 h of admission; daily up to day 8/discharge/death	High	Cerebral malaria patients (*n* = 160 children, ages 3 months–18 years) and severe malaria without neurologic symptoms (*n* = 155 children)	Democratic Republic of the Congo	NP	84
	Lung	Lung aeration patterns, interstitial syndrome, consolidation, acute respiratory distress syndrome (ARDS) (Kigali definition)	Lung ultrasound (LUS)	Normal LUS: 7% fatality; interstitial syndrome: 55% fatality; ARDS: 90% fatality; LUS + dyspnea and oxygen desaturation (SpO_2_)/fraction of inspired oxygen (FiO_2_) area under the receiver operating characteristics curve (AUROC) 91%	<15 min	High	Febrile patients (*n* = 102 adults, ages > 12 years)	Chittagong, Bangladesh	NP	85
	Inferior vena cava (IVC), lungs	IVC collapsibility index (IVC-CI); B-pattern lung ultrasound	Point-of-care ultrasound (POCUS) using IVC and lung ultrasound	IVC-CI ≥ 50% correlated with signs of hypovolemia (sunken eyes *r* = 0.35, prolonged capillary refill time *r* = 0.35); B-pattern associated with dyspnea, crepitations, and SpO_2_ ≤ 94%	<15 min	High	Febrile patients (*n* = 48) and healthy controls (*n* = 62)	Lambaréné, Gabon	NP	89
	Patient (brain circulation)	Cerebral blood flow velocity patterns (phenotypes: hyperaemia, low flow, microvascular obstruction, vasospasm, isolated posterior high flow)	TCD	Overall: 68% good, 13% mortality; hyperaemia 77%, low flow 42% (30% mortality); good outcome with higher transient hyperemic response ratio (THRR)	First scan within 4 h of admission; daily monitoring up to day 8 or discharge	High	Febrile patients (*n* = 174, children ages 6 months–12 years)	Blantyre, Malawi	NP	90
Photoacoustics	Human transdermal blood (wrist/earlobe); *Anopheles* mosquitoes	Hemozoin; all *Plasmodium* species	Photoacoustic imaging (hybrid vapor nanobubble; H-VNB): pulsed laser (532 nm, 10 μJ, 200 ps, 36 mJ cm^−2^); integrated optical fiber-acoustic sensor probe	Limit of detection (LOD): *in vivo* 0.00034%; patient 0.3–2% parasitemia; no false positives	20 s	High	1 patient; healthy volunteers; infected/uninfected mosquitoes	USA; lab study	NP	81
	Finger (capillary blood *in vivo*)	Hemozoin; all *Plasmodium* species	Photoacoustic spectroscopy: infrared (IR) laser 980 nm with 0.3 T magnet; S4111-16R photodetector	NP/NP/95% (38/40)	NP	Moderate	40 patients (male only)	Sudan; lab study	NP	83
	Finger (*in vivo*)	Hemozoin; *P. falciparum*	Photoacoustic imaging (Cytophone): pulsed laser (1064 nm); focused ultrasound transducer array; real-time AI signal analysis	90%/69%/73%, area under the receiver operating characteristic curve (ROC-AUC) 0.84 *vs.* microscopy; comparable to quantitative polymerase chain reaction (qPCR)	<5 min	Moderate	30 adults (Cameroon): *n* = 10 safety, *n* = 20 performance; 94 measurements	Cameroon	Yes, possible	92
Spectroscopy/imaging	Fingernail	Hemozoin	Magneto-optic spectroscopy: 658–660 nm laser; polarization modulation 88–100 kHz	26.7%/80.6%/63%	∼20 s	Moderate	Suspected malaria patients (*n* = 46, teenagers and adults)	Mbita, Kenya	NP	77
	Buccal mucosa	Endothelial glycocalyx integrity (PBR), perivascular hemorrhages, parasite sequestration	Handheld Cytocam-Incident dark-field (IDF) imaging device: green light-emitting diode (LED) (525 nm); real-time video microscopy; automated PBR calculation	Significantly increased PBR in severe malaria (*p* < 0.0001); ∼50% showed perivascular hemorrhages; visualization of late-stage parasite sequestration; accuracy NP	∼5–10 min	Moderate	Pediatric patients: severe malaria (*n* = 69), uncomplicated malaria (*n* = 12), non-malarial fever (*n* = 7), healthy controls (*n* = 31)	Mwanza region, Tanzania	NP	67
	Skin (ear, finger, arm)	Malaria parasite-associated absorption peaks (hemozoin, Hb, parasite proteins/lipids)	Hand-held NIRvascan spectrometer (NIRS): 900–1700 nm, machine learning analysis (NN)	Ear: 100%/85%/92%; finger: 72%/69%/70%; arm: 59%/85%/72%	∼5–10 s	High	Symptomatic patients (*n* = 60; *P. vivax* = 20, *P. falciparum* = 7)	São Gabriel da Cachoeira, Brazil	NP	73
	Urine	Amino acids and metabolites (cystine, lysine, tyrosine, creatinine)	Fourier-transform infrared (FTIR) spectrophotometer (buck converter 530, 4000–600 cm^−1^)	Principal component analysis (PCA) distinguished malaria *vs.* controls; cystine significantly higher (*p* = 0.012)	∼3 min per sample (IR)	Moderate	112 participants (*n* = 41 malaria-positive, *n* = 71 controls; ages 3–50 years)	Mbarara City, Uganda	NP	48
	Urine	*P. vivax* parasite-derived proteins (28 proteins, including rifin, profilin, single-stranded DNA-binding protein)	SDS-PAGE + in-gel/in-solution trypsin digestion; LC-MS/MS; gene ontology and functional enrichment analysis (DAVID, PANTHER, g: profiler)	28 *P. vivax*-specific proteins identified exclusively in infected samples; 252 human proteins unique to infected samples; no diagnostic sensitivity/specificity reported (biomarker discovery study)	NP	High	*n* = 8 (4 *P. vivax*-positive, 4 healthy controls; ages 20–50 years)	Ajmer, Rajasthan, India	NP	50

a NP: not provided.

Field-based studies extended VOC analysis to skin-emitted profiles and evaluated detection robustness under endemic conditions. De Moraes *et al.*^75^ analyzed volatile emissions from the feet and arms of Kenyan schoolchildren. Rather than relying on a single compound, their GC-MS plus machine-learning workflow identified recurring multivariable skin-odor patterns and achieved up to 95% sensitivity with 77% accuracy from foot samples and 92% sensitivity with 80% accuracy from arm samples. Importantly, the approach reached 100% sensitivity for asymptomatic infections, indicating that this workflow can detect hidden reservoirs of transmission. To further evaluate detection specificity, Pulido *et al.*^74^ compared skin VOC profiles of malaria-positive children with those suffering from malaria-like febrile conditions. Their predictive models achieved 100% accuracy in febrile children and 75% across broader symptomatic groups, supporting the value of pattern-level chemical signatures over repeated interpretation of individual compound names. Malaria VOC detection using GC-MS has yielded encouraging classification results but faces several limitations. VOC profiles vary with diet, co-infections, host genetics, environmental factors, and *Plasmodium* species, leading to inter- and intra-individual variability that complicates biomarker standardization.^68,70,74,75^ Differences in sample collection and analytical workflows further result in inconsistent compound panels across studies. GC-MS also requires specialized instrumentation, technical expertise, and controlled environments, limiting routine field deployment.^68,69^ Despite these challenges, VOC profiling offers clear advantages, including its noninvasive format and capacity to differentiate malaria from other febrile illnesses.^72,74,75^ Future progress will rely on multicenter validation, standardized methodologies, and translation into portable platforms such as electronic noses or AI-driven sensors for field-deployable screening.^68–70^

### 3.2. Acoustic, photoacoustic, and optical systems

Acoustic, photoacoustic, and optical methods provide noninvasive and real-time sensing capabilities by detecting malaria-associated alterations in tissue structure, blood flow, or parasite-derived optical absorption without requiring blood collection (Table 3). Ultrasound imaging has been widely used to assess organ-level abnormalities, particularly in severe or cerebral malaria, using external probes. In parallel, photoacoustic and spectroscopic methods utilize light-tissue interactions to detect parasite-specific signatures (*e.g.*, hemozoin) through the skin or other accessible interfaces. Accordingly, ultrasound is best understood as an adjunctive acoustic tool for detecting disease-associated physiological or organ-level changes, whereas photoacoustic and optical approaches are more directly linked to pigment- or spectrum-based detection and, in some cases, parasite-burden estimation. This section reviews recent advances in ultrasound, photoacoustic, and spectroscopic strategies for malaria detection.

#### 3.2.1. Ultrasound

**Fig. 6:**
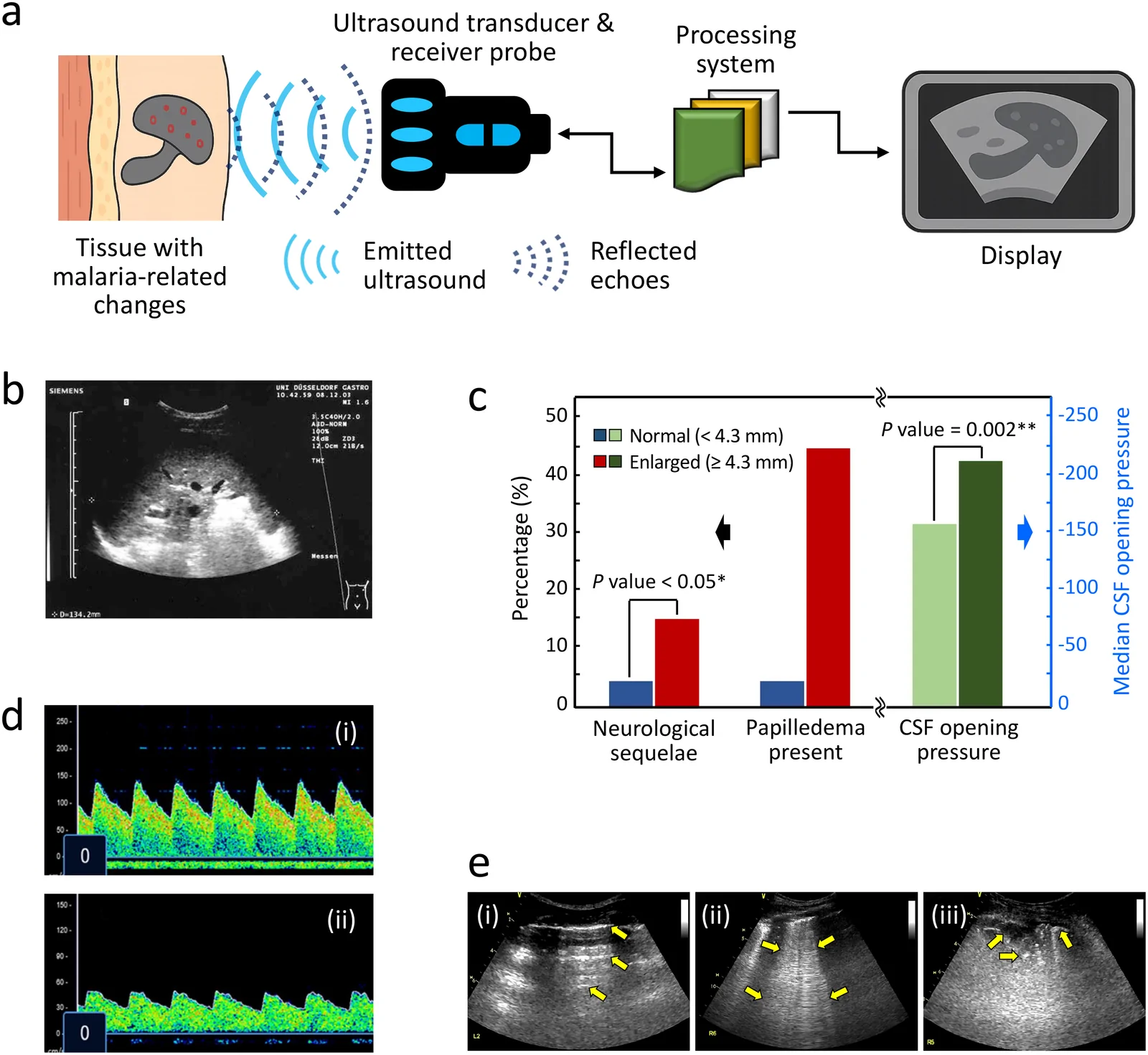
Ultrasound-based malaria detection. (a) Schematic illustration of ultrasound imaging. Emitted ultrasound waves are reflected from tissue with malaria-related changes, processed, and displayed to visualize anatomical or physiological alterations. Original illustration created by the authors. (b) Ultrasonographic scan of the spleen in a patient with hyperreactive malarious splenomegaly, showing characteristic splenic enlargement^76^ [adapted from ref. 76 with permission from Springer Nature; © 2004 Springer Nature.]. (c) Optic nerve sheath diameter (ONSD) measurements in children with cerebral malaria.^79^ Patients with enlarged ONSD (≥4.3 mm) showed higher frequencies of neurological sequelae and papilledema, and significantly elevated cerebrospinal fluid (CSF) opening pressure, supporting ONSD ultrasound as a noninvasive marker of raised intracranial pressure (*Fisher exact test; **Student *t*-test) [adapted from ref. 79 with permission from the American Society of Tropical Medicine and Hygiene; © 2012 American Society of Tropical Medicine and Hygiene.]. (d) Transcranial Doppler (TCD) ultrasound waveforms.^90^ (i) Representative middle cerebral artery (MCA) waveform from a healthy child, showing normal systolic and diastolic flow velocities. (ii) TCD waveform from a child with cerebral malaria, characterized by globally diminished systolic, diastolic, and mean velocities, indicating reduced cerebral perfusion and an elevated risk of adverse outcomes [adapted from ref. 90 under the terms of the CC BY 4.0 license; © 2022 The Author(s).]. (e) Lung ultrasound patterns (LUS).^85^ (i) A-lines: reverberation artifacts from normally aerated lungs. (ii) B-Pattern: multiple coalescent B-lines indicating pulmonary edema. (iii) C-Pattern: signs of pulmonary consolidation, including pleural line interruption and echogenic punctiform lesions with air bronchograms [adapted from ref. 85 under the terms of the CC BY 4.0 license; © 2018 Leopold *et al.*].

Ultrasound is a noninvasive imaging modality that generates real-time images of internal structures by transmitting high-frequency sound waves and reconstructing the reflected echoes (Fig. 6a). In malaria, ultrasound detects infection-related alterations in host organs, including the brain, eye, liver, spleen, kidney, and lungs.^93^ Depending on the application, clinicians may measure optic nerve sheath diameter to infer raised intracranial pressure,^78,79^ assess cerebral blood flow in the middle cerebral artery,^84,90^ or evaluate hepatosplenomegaly and renal abnormalities through abdominal imaging.^76,80,82^ Pulmonary manifestations, including edema and consolidation, can also be identified using lung ultrasound.^85,89^ These ultrasound findings, reflecting perfusion abnormalities, inflammatory changes, and organ enlargement, serve as surrogate markers of severity rather than parasite presence.^93^ Abdominal imaging remains the most established application. Richter *et al.* reported splenomegaly in 82.8% of patients, including 100% of children, and persistence at day 21 in all recrudescence or relapse cases compared with 59.3% of cured patients, although the authors concluded that its practical utility was limited^76^ (Fig. 6b). In a large pediatric cohort, Laman *et al.* found hepatomegaly in 71% and splenomegaly in 64%, underscoring its detection value in endemic populations.^82^ Optic nerve sheath diameter (ONSD) ultrasound has emerged as a practical tool for assessing intracranial pressure. Beare *et al.* reported raised intracranial pressure in 49% of children with cerebral malaria using an enlarged ONSD threshold of at least 4.3 mm, with enlarged ONSD associated with neurological sequelae and papilledema^79^ (Fig. 6c). Similarly, Murphy *et al.*^78^ observed increased ONSD in about one third of patients, reaching 100% in cerebral malaria. Elevated middle cerebral artery (MCA) velocity was noted in 25% of children with severe anemia, and splenomegaly was detected in 24% by ultrasound compared with 48% by palpation.

Transcranial Doppler (TCD) ultrasound, performed through the temporal bone window, provides valuable information on cerebral hemodynamics. O'Brien *et al.* reported impaired autoregulation in 80% of pediatric cerebral malaria cases, with survivors exhibiting significantly higher transient hyperemic response ratio (THRR) values than nonsurvivors (1.09 *vs.* 0.98, *p* ≤ 0.001).^84^ In a follow-up study, O'Brien *et al.* identified distinct flow phenotypes, including a “low flow” pattern in 19% of cases, which was strongly associated with poor outcomes^90^ (Fig. 6d). These results highlight the prognostic potential of TCD in understanding cerebral malaria pathophysiology. Beyond cerebral imaging, ultrasound has been applied to systemic complications. Lung ultrasound (LUS) revealed malaria-associated pulmonary changes, with Leopold *et al.* detecting B-line patterns in 48% of patients and consolidation in 17%^85^ (Fig. 6e). Renal ultrasound demonstrated measurable alterations, as Atalabi *et al.* observed kidney enlargement of up to 0.41 cm and increased renal volume, while Pugliese *et al.* found that an inferior vena cava collapsibility index ≥50% correlated with hypovolemia and concurrent LUS B-patterns with dyspnea and oxygen desaturation (SpO_2_ ≤ 94%).^80,89^

The application of ultrasound in malaria detection is subject to significant limitations. Splenomegaly or pulmonary edema are not specific to malaria and may also arise from other infectious or inflammatory conditions.^76,85,93^ Detection accuracy is influenced by operator skill, and reference thresholds (*e.g.*, ONSD, spleen size) vary with age and body size.^79,82^ Moreover, ultrasound cannot directly detect parasites, restricting its role to assessing complications and severity rather than confirming infection.^76,78^ Nonetheless, its ability to noninvasively evaluate malaria-related pathology in real time, particularly in pediatric and cerebral presentations, makes ultrasound a valuable adjunct to traditional diagnostics. With expanding access to portable devices and training programs, ultrasound may become an integral component of decentralized malaria care and triage systems.^93^

#### 3.2.2. Photoacoustic-based methods

**Fig. 7:**
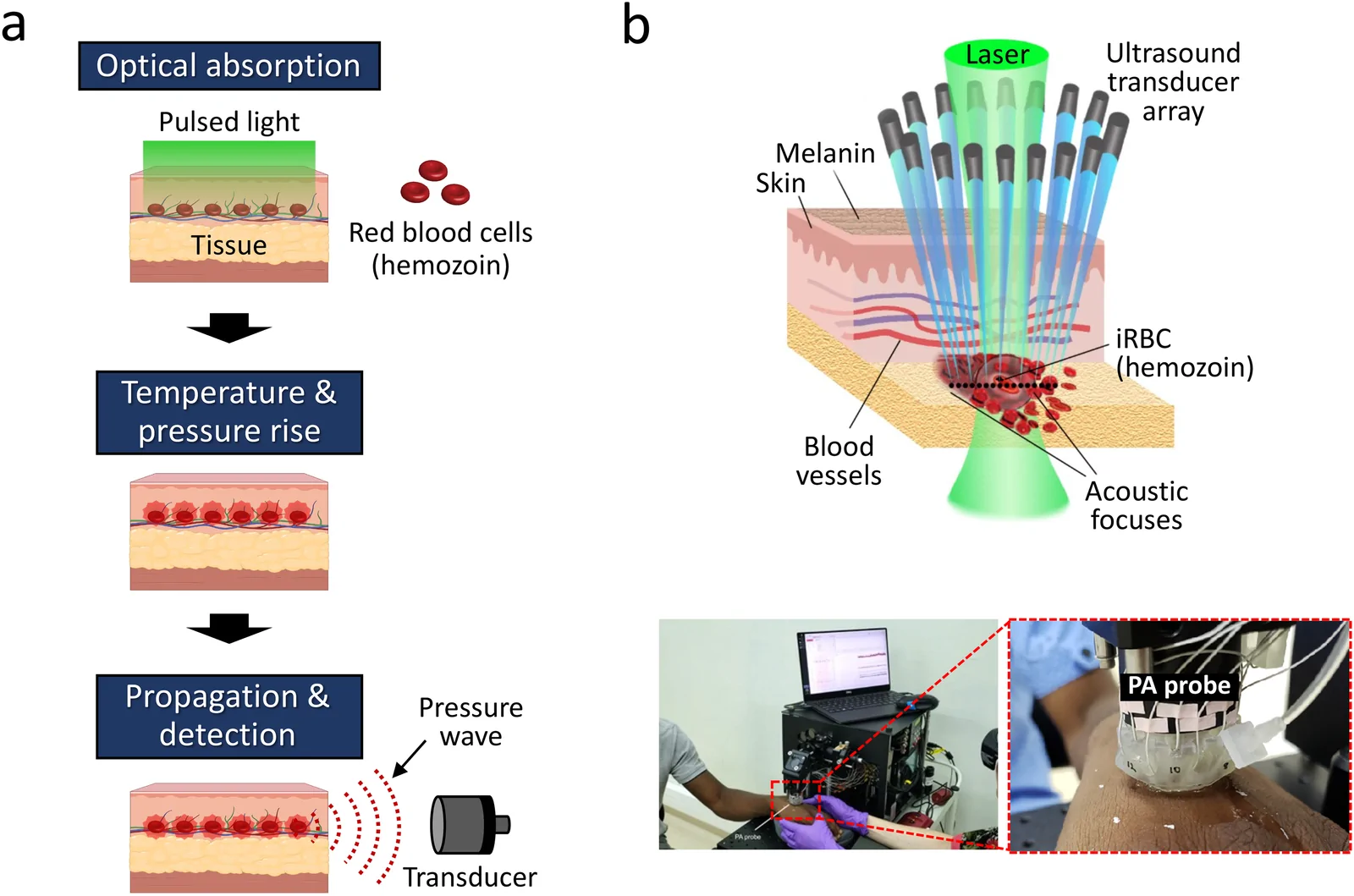
Photoacoustic-based malaria detection. (a) Schematic illustration of the photoacoustic detection mechanism. Pulsed laser light is absorbed by hemozoin within *Plasmodium*-infected red blood cells (iRBCs), generating localized thermoelastic expansion and resulting in acoustic wave emission. These waves are captured by an ultrasound transducer through the skin. Original illustration created by the authors using https://BioRender.com. (b) *In vivo* transdermal detection of malaria using the Cytophone system.^92^ Top: Diagram of the arm-mounted ultrasound array. Bottom: Photos of the corresponding laser-acoustic system. PA: photoacoustic [adapted from ref. 92 with permission from Springer Nature; © 2024 The Author(s).].

Photoacoustic methods detect malaria by converting pulsed laser light into acoustic waves *via* hemozoin, a crystalline byproduct formed during hemoglobin digestion in *Plasmodium*-infected red blood cells (iRBCs).^81,92,119^ Hemozoin absorbs light strongly in the visible and near-infrared ranges, generating rapid thermoelastic expansion that produces acoustic signals detectable through the skin (Fig. 7a). This label-free mechanism avoids the need for reagents or blood sampling, enabling fully noninvasive detection. Two main approaches exist: photoacoustic imaging (or transdermal flow cytometry), which identifies signals from individual iRBCs in peripheral vessels,^81,92,119^ and photoacoustic spectroscopy, which measures signal intensity across wavelengths to infer hemozoin presence.^83,119^ These methods are typically applied to areas with superficial vasculature (*e.g.*, the wrist, fingertip, or earlobe) and are suitable for real-time, *in vivo* detection. Lukianova-Hleb *et al.*^81^ developed a transdermal photoacoustic system that detected hemozoin-generated vapor nanobubbles in wrist and earlobe vessels. Using a 532 nm pulsed laser, the platform distinguished malaria-positive signals within 20 s and achieved a detection limit of approximately 0.3% parasitemia. Yadem *et al.*^92^ advanced this concept with an arm-mounted handheld flow cytometry-based Cytophone capable of detecting individual iRBCs in wrist vessels. In a longitudinal clinical study, the device demonstrated 90% sensitivity and 69% specificity relative to microscopy, with distinctive acoustic traces observed in malaria-infected individuals (Fig. 7b). In contrast, Osman *et al.*^83^ introduced a low-cost spectroscopy-based platform using a 980 nm laser and a photodetector applied to fingertip vessels, which achieved 95% accuracy in a cohort of 40 patients, although no signals were obtained in female participants. The original report did not provide sufficient evidence to assign a specific cause for this sex-specific result; plausible contributors include anatomical or perfusion differences at the fingertip, probe coupling or alignment, skin and tissue optical properties, cohort imbalance, or technical thresholding effects. This observation requires explicit replication before being interpreted as a biological sex effect.

Several limitations currently constrain photoacoustic malaria detection. Endogenous absorbers (*e.g.*, melanin and hemoglobin) can reduce signal specificity, prompting exploration of red and near-infrared excitation bands that offer improved contrast with hemozoin.^102,120^ Flow cytometry-based systems also require precise alignment with superficial vessels, which may constrain usability in field conditions.^92^ Device costs remain high due to the reliance on specialized laser and ultrasound transducer components.^83,120^ Nevertheless, photoacoustic-based methods offer notable advantages: they are label-free, exploit hemozoin that is universally present in blood-stage parasites, and enable real-time, noninvasive detection. Advances in miniaturization, cost reduction, and automated signal processing are expected to enhance their feasibility for field deployment and large-scale screening programs.^92,119,120^

#### 3.2.3. Optical spectroscopy and imaging

Hemozoin has been investigated as a potential biomarker for malaria using optical spectroscopy and imaging. Optical spectroscopy and imaging enable quantitative assessment of how biological tissue absorbs and scatters light within defined spectral ranges.^120–122^ In the visible to near-infrared (NIR; 400–2500 nm) region, endogenous chromophores (*e.g.*, hemoglobin and hemozoin) generate distinctive absorption and scattering profiles.^26,73,120^ Importantly, hemozoin exhibits broadband absorption from the visible to NIR spectrum and represents a key biomarker for optical detection.^77^ Spectroscopy-based platforms typically measure reflectance or transmittance from accessible body sites, including the ear, finger, arm, lip, and buccal mucosa;^67,73,77^ however, they have also been adapted to alternative matrices (*e.g.*, urine).^48,50^ Magneto-optic probes exploit the paramagnetic properties of hemozoin, aligning its crystals with an external magnetic field and detecting polarization-dependent changes in transmitted light, thereby enabling rapid *in vivo* detection.^77^

**Fig. 8:**
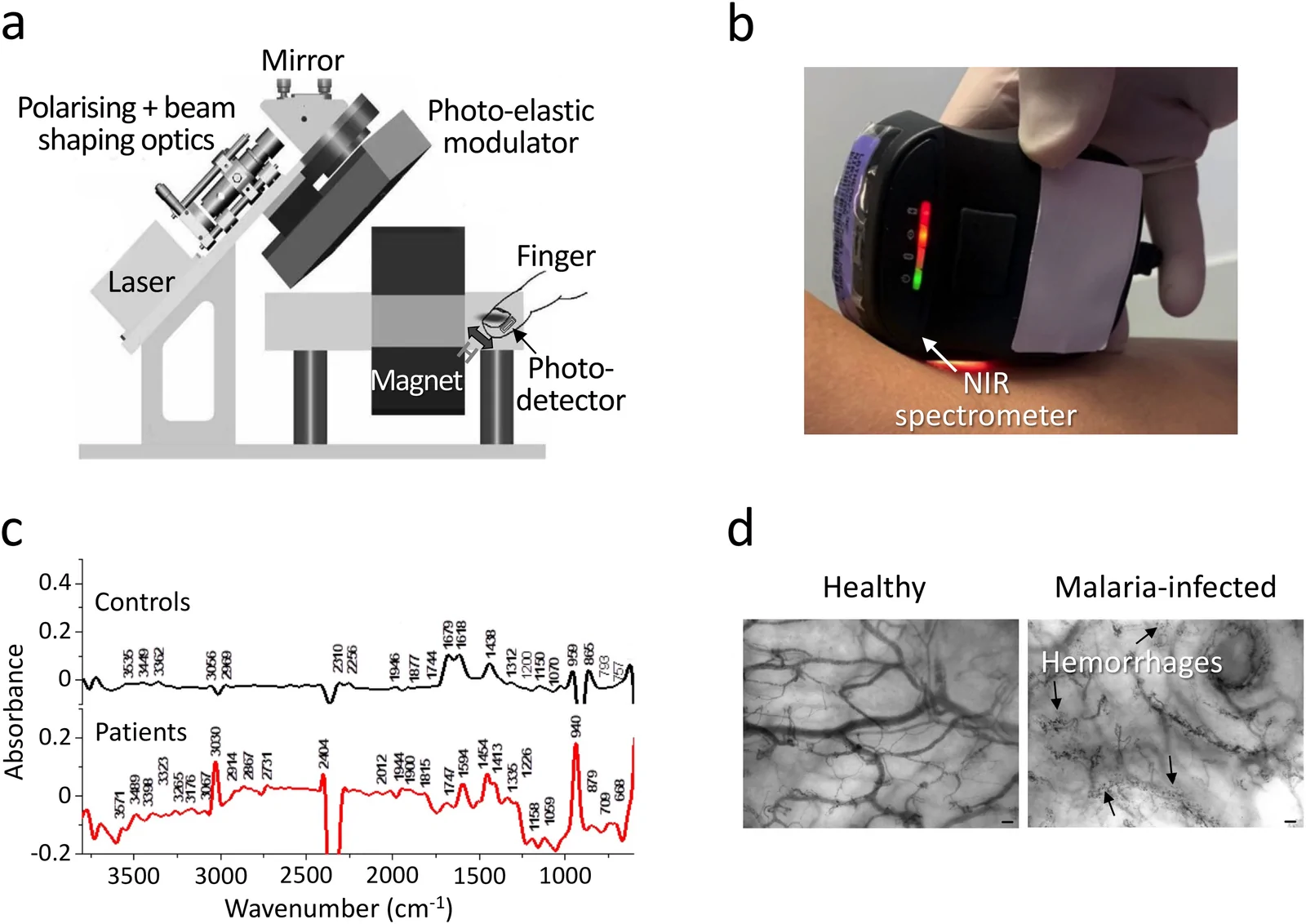
Optical spectroscopy and imaging methods for noninvasive malaria detection. (a) Magneto-optic device for *in vivo* hemozoin detection.^77^ The system includes a laser source, polarizing and beam-shaping optics, a photoelastic modulator, an applied magnetic field, and a photodetector for fingertip measurement [adapted from ref. 77 with permission from IEEE; © 2010 IEEE.]. (b) Near-infrared (NIR) spectroscopy (NIRvascan) for parasite burden assessment.^73^ Photographs showing noninvasive scanning of the upper arm with a handheld spectrometer [adapted from ref. 73 with permission from Oxford University Press; © 2022 Oxford University Press.]. (c) Fourier-transform infrared (FTIR) spectroscopy using urine sample.^48^ Average IR spectra from controls (black) and malaria patients (red) [adapted from ref. 48 under the terms of the CC BY 4.0 license; © 2024 The Author(s).]. (d) Incident dark-field (IDF) imaging of the buccal microcirculation.^67^ Left: Representative images showing healthy vessels *versus* malaria-associated perivascular hemorrhages. Right: Quantification of hemorrhage density across groups [adapted from ref. 67 with permission from the American Society for Microbiology; © 2020 American Society for Microbiology].

Light-tissue interaction-based methods were first studied by Newman *et al.*,^77^ who developed a noninvasive magneto-optic (NIMO) fingertip probe that detects hemozoin by magnetic alignment and polarization-dependent absorption (Fig. 8a). Phantom tests demonstrated a detection limit of below 0.02 μg mL^−1^ β-hematin (∼33 parasites per μL), with measurements completed in ∼10 s. A prototype operating at 660 nm with a 0.5 T field confirmed linear responses from intracellular hemozoin, but a Kenyan field trial (*n* = 46) achieved only 26.7% sensitivity and 80.6% specificity, constrained by finger positioning and blood-flow artifacts. Garcia *et al.*^73^ advanced this principle with a handheld NIRvascan spectrometer (Fig. 8b). By scanning ear, finger, and arm sites, they identified a consistent 960 nm absorption peak linked to parasitemia. Neural network analysis of ear spectra yielded 100% sensitivity, 85% specificity, and 92% accuracy, detecting infections as low as 300–500 parasites per μL within 10 s using a 136 g reagent-free device.

Beyond blood-associated signals, body-fluid and mucosal imaging have also been investigated. Birungi *et al.*^48^ applied Fourier-transform infrared (FTIR) spectroscopy on urine (Fig. 8c), revealing metabolic perturbations in the mid-infrared range (4000–600 cm^−1^). Distinct spectral peaks linked to amino acids (1618, 1679 cm^−1^) and ammonium (3030 cm^−1^) differentiated malaria patients from controls, with principal component analysis (PCA) clustering and liquid chromatography-tandem mass spectrometry (LC-MS/MS) validation showing significant cystine elevation (*p* = 0.012) and increases in lysine and tyrosine. Tripathi *et al.*^50^ identified 28 parasite-derived proteins in urine from *P. vivax*-infected patients using LC-MS/MS, including rifin-like protein, profilin, and single-stranded DNA-binding protein, supporting the feasibility of urine as a source of parasite antigens for noninvasive proteomic detection. At the tissue level, Lyimo *et al.*^67^ employed Cytocam incident dark-field (IDF) imaging of the buccal mucosa (Fig. 8d). Severe malaria patients exhibited an increased perfused boundary region (median ∼4.5 μm *vs.* 3.5 μm in controls, *p* < 0.0001), a ∼50% prevalence of perivascular hemorrhages, and elevated plasma levels of hyaluronic acid and heparan sulfate, correlating with glycocalyx loss.

Optical spectroscopy and imaging for malaria detection offer distinct advantages but also face challenges. Signal fidelity can be affected by skin pigmentation, tissue heterogeneity, and motion artifacts,^67,77^ while reliable correlations between spectral features and parasite density remain difficult at low parasitemia.^48,73^ Environmental interference, physiological variability, and the lack of standardized protocols further limit reproducibility across sites.^26,68^ Nevertheless, their rapid, label-free nature and compatibility with portable platforms support further translational evaluation.^26,73,120^ Recent advances, including miniaturized microelectromechanical systems (MEMS)/complementary metal–oxide–semiconductor (CMOS) spectrometers and wearable NIRS devices capable of continuous, wireless monitoring with on-device machine learning, highlight a clear pathway toward point-of-care deployment.^120,123^ Together with improved algorithms for spectral noise reduction and parasitemia quantification, these innovations could enable early detection of asymptomatic carriers and integration into broader malaria control programs.^26,120,123^

### 3.3. Digital and connected health platforms for noninvasive malaria detection

Recent advances in mobile health and wearable technologies have opened new pathways for noninvasive malaria detection. Digital and connected platforms allow the acquisition of physiological and anatomical signals using smartphones, smartwatches, and other portable devices without the need for blood sampling.^124–126^ These tools can support continuous monitoring, early risk assessment, and scalable deployment in field settings.^124,125^ Unlike conventional detection methods that rely on centralized infrastructure, digital platforms can operate autonomously and remotely, making them particularly suited for low-resource or high-transmission environments.^124^ By capturing signals from accessible sites (*e.g.*, the eye, skin, or wrist) and analyzing them with machine learning and AI, these systems can detect subtle physiological changes associated with malaria infection.^86,88^ This section introduces emerging digital and connected approaches, including wearable sensing of vital signs and smartphone-based imaging, to enable early, user-centered, and scalable malaria surveillance.

#### 3.3.1. Integration of connected wearable devices into digital malaria detection

Wearable devices are increasingly integrated into digital health systems to enable continuous, noninvasive monitoring of physiological signals relevant to malaria detection, as summarized in Table 4. Parameters (*e.g.*, heart rate, skin temperature, and body acceleration) can reflect malaria-associated manifestations including fever, fatigue, and altered cardiovascular responses. Recent progress in miniaturized sensors, wireless connectivity, and edge computing has expanded the role of wearables beyond activity tracking toward health monitoring applications that require minimal user intervention.

**Table 4:** Summary of studies on digital and connected platforms for noninvasive malaria detection

Detection method	Sample/Signal source	Platform detail	Machine learning (ML)	Performance	Low-resource field usability	Research population	Research area	Detect asymptomatic infection	Ref.
Wearable sensors	Physiological parameters: heart rate, body temperature, estimated blood glucose	Wearable sensors (heart rate (HR) sensor, DHT11 thermometer) + Arduino MEGA 2560 + Raspberry Pi 3B+ for data processing and display	Logistic regression (naïve Bayes, SoftMax function)	High correlations (glucose, *t* = 0.9934, heart rate, *r* = 0.9512); low correlation (body temperature, *r* = 0.0925)	Low	Tested on malaria patients and healthy individuals (small sample size, not specified)	India	No	86
IoT-based real-time wearable sensors	Wearable and smartphone-collected symptom data (15 features: fever, chills, headache, vomiting, diarrhea, anemia, jaundice, hypoglycemia, *etc.*)	IoT framework: wearable sensors → edge (fog) computing → cloud infrastructure → physician interface	Compared machine learning (ML) models (support vector machine (SVM), neural network (NN), k-nearest neighbor (KNN), Naïve Bayes); SVM best: 98% train, 96% test acc., area under curve (AUC) 95%	Testing accuracy: 96% (SVM), 93% (NN), 91% (KNN), 62% (naive Bayes)	Low	Dataset of 1011 cases (*n* = 663 negative, *n* = 348 confirmed malaria)	Nigeria	NP^a^	91
Wearable sensors	Physiological signals: heart rate, skin temperature, body acceleration	Samsung Gear S3 smartwatch + Galaxy S7 smartphone	Bayesian model (“2B-Healthy”) detecting aberrant heart rate patterns adjusted for circadian rhythm & activity	Sensitivity 78% (7/9 parasitemic), ∼67% detected 6 days before parasitemia; specificity 75% (6/8 controls); false positive rate 6% per week	Low	12 Controlled human malaria infection (CHMI) subjects (10 analyzable, 9 parasitemic) + 9 controls, healthy adults 18–50 years	USA (controlled CHMI study, Naval Medical Research Center (NMRC)/Walter Reed Army Institute of Research (WRAIR))	Yes, detected infection before parasitemia and symptoms	88
Mobile imaging	Palpebral conjunctiva (inner eyelid) photos, 4302 images from 405 children (ages 5–15 years)	Smartphones (Samsung Galaxy S22, Google Pixel 6)	Radiomic features + random forest + neural network; mask region-based convolutional neural network (Mask R-CNN) segmentation; standardized smartphone photo protocol	AUC 0.76 (95% CI: 0.68–0.84)	High	Asymptomatic children (*n* = 405, school-age, 5–15 years)	Gisagara District in Rwanda	Yes, stratified asymptomatic malaria risk	94

a NP: not provided.

**Fig. 9:**
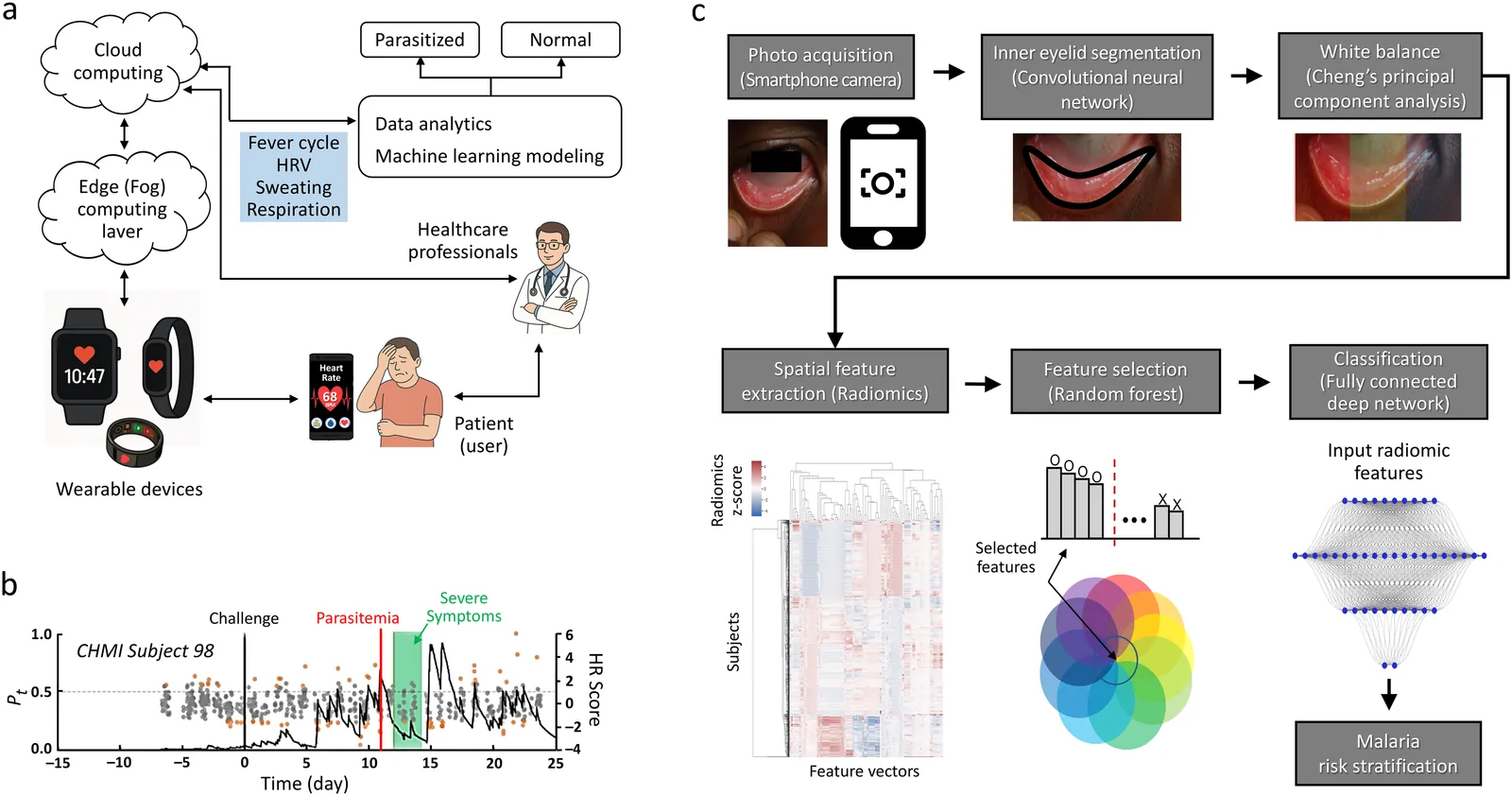
Digital and connected platforms for noninvasive malaria detection using wearable sensing and smartphone-based imaging. (a) Schematic illustration of a connected wearable platform integrating real-time physiological monitoring, edge computing, and cloud-based analytics.^91^ Physiological signals (*e.g.*, heart rate, heart rate variability (HRV), fever cycle, sweating, and respiration) are analyzed *via* machine learning to differentiate infected from normal states, enabling timely feedback to users and clinicians. Created by the authors using https://BioRender.com and adapted from ref. 91 with permission from Springer Nature; © 2024 The Author(s). (b) Longitudinal monitoring from a controlled human malaria infection (CHMI) study.^88^ Predicted malaria probability (*P*_*t*_) rises ahead of both parasitemia and clinical symptom onset, reflecting early discriminatory potential closely linked to heart rate (HR) dynamics [adapted from ref. 88 with permission from IEEE; © 2022 IEEE.] (c) Radiomics-based machine learning pipeline for malaria risk stratification using smartphone-acquired conjunctiva images.^94^ Inner eyelid images were processed with deep learning-based segmentation, white balance correction, and radiomic feature extraction for classification, achieving discrimination against rapid diagnostic test (RDT)-confirmed infection. This demonstrates the feasibility of contactless, infrastructure-free smartphone imaging for community-level malaria screening [adapted from ref. 94 with permission from Springer Nature; © 2025 The Author(s)].

Ravi e*t al.*^86^ developed an Internet of Things (IoT)-based system that uses noninvasive inputs (*e.g.*, body temperature, heart rate, and estimated glucose) to predict malaria using logistic regression. The model showed strong correlations (*r* = 0.9934 for glucose, 0.9512 for heart rate) and demonstrated high accuracy in tests involving both healthy and malaria-positive individuals without blood sampling. However, body temperature showed only a weak correlation with malaria status (*r* = 0.0925), whereas glucose and heart rate demonstrated stronger relationships. Ayalew *et al.*^91^ reported a real-time malaria monitoring framework using wearable IoT sensors (Fig. 9a). The system collected symptom-related data (*e.g.*, fever, exhaustion, anemia, jaundice) through connected sensors, processed them *via* edge computing, and transmitted results to a cloud platform with physician integration. Infection status was classified using machine learning models, including support vector machine (SVM), neural network (NN), k-nearest neighbor (KNN), and naïve Bayes, with the best-performing approach achieving up to 96% test accuracy and 95% AUC for SVM. In addition, Chaudhury *et al.*^88^ demonstrated that commercially available smartwatches can support noninvasive malaria detection (Fig. 9b). In a controlled human malaria infection (CHMI) study, participants wore Samsung Gear S3 smartwatch, and the physiological data were analyzed using a Bayesian algorithm to estimate infection probability. The system achieved 78% sensitivity, detecting 67% of infections before parasitemia, with a 6% false-positive rate for a median of ∼6.4 days before the first detection of parasitemia (quantitative PCR reference).

The hierarchical architecture of these studies linking wearable sensors, edge devices, cloud analytics, and healthcare providers illustrates how connected wearables, combined with AI-driven analytics, can enable real-time, noninvasive malaria risk detection and support timely clinical decision-making.^88,91^ However, wearable-based digital platforms for malaria detection face several important limitations. Most notably, device adoption remains low in malaria-endemic regions, with smartwatch penetration under 5% in many low-income countries and access limited primarily to urban populations.^124^ Infrastructure gaps, including unreliable connectivity, limited electricity, and high device costs, further constrain deployment.^124,127^ Additionally, reliance on nonspecific physiological signals (*e.g.*, heart rate and body temperature) may compromise detection specificity.^86,88^ Sensor performance can also be affected by environmental conditions, and integrating AI-driven tools into existing health systems poses logistical and regulatory challenges.^124,127^ Cultural acceptance and user adherence may limit uptake in some settings.^124^ Addressing these barriers will require not only technical development, but also improvements in affordability, infrastructure, and community trust to enable real-world use of wearable detection in high-burden regions. These digital platforms may also support more equitable deployment by enabling decentralized monitoring in resource-limited settings, although disparities in device access and digital infrastructure remain important considerations.^9^

#### 3.3.2. Smartphone-based conjunctiva imaging for digital malaria detection

Advances in smartphone imaging (*i.e.*, digital photography) and on-device machine learning have enabled low-cost, scalable solutions for clinical screening using photographs of accessible body sites.^128–131^ The palpebral conjunctiva, located inside the lower eyelid, offers a noninvasive window into microvascular and hematologic status, including signs of anemia, a common comorbidity in malaria.^132–135^ Malaria infection causes changes to microvascular properties. For example, *P. falciparum* (distinct from other species) infects red blood cells, which circulate before adhering to endothelial cells and becoming sequestered in the vasculature, triggering microvascular alterations that contribute to severe disease.^136–138^ Importantly, the palpebral conjunctiva contains dense microvascular networks that offer a rich source of vascular information.^131,132,134,135,139,140^ In this respect, smartphone imaging of the conjunctiva is contact-free, requires no consumables, and can be deployed in resource-limited settings without clinical infrastructure. The increasing penetration of smartphones in malaria-endemic regions provides a scalable and cost-effective pathway to expand detection capacity, reducing reliance on specialized hardware and trained personnel while supporting surveillance and care in resource-limited settings.^141–143^ Hong *et al.*^94^ recently reported the first demonstration of smartphone conjunctiva imaging for malaria detection, marking a significant advance in noninvasive digital detection (Fig. 9c). In this study, over 4000 conjunctiva images were acquired from 405 school-aged participants in Rwanda using standard Android smartphones under ambient light, reflecting real-world field conditions. A novel pipeline integrating Mask region-based convolutional neural network (MASK R-CNN) segmentation, radiomic feature extraction, and neural network classification achieved an AUC of 0.76 (95% CI: 0.68–0.84) against RDT-confirmed infection. Unlike prior noninvasive approaches that relied on physiological signals from wearables or molecular assays from saliva and urine, this vision-based method enables contactless, infrastructure-free community screening using ubiquitous consumer devices. Its design demonstrates a scalable deployment pathway in malaria-endemic settings, particularly for pediatric populations. While challenges including lighting variability remain, this work establishes a new paradigm for mobile, AI-powered imaging in malaria surveillance and supports continued evaluation of digital platforms in advancing noninvasive detection. Such approaches may enhance accessibility by leveraging widely available mobile devices; however, variability in smartphone access, connectivity, and data quality highlights the need to address digital divides and ensure equitable implementation across diverse populations.^9^

#### 3.3.3. AI-enabled noninvasive malaria detection

Machine learning and AI have emerged as a transformative force in diagnostic medicine, offering advanced capabilities to process, interpret, and integrate complex biological, physiological, and environmental data.^139,144–147^ In malaria detection, AI-based approaches, encompassing machine learning, deep learning, and statistical modeling, have been applied to various detection platforms, including microscopy, nucleic acid amplification tests (*e.g.*, PCR, LAMP, or NASBA), biosensors, image-based parasite identification, and laboratory-based immunoassays.^21,148–153^ These studies demonstrate how machine learning and AI can amplify weak biomarker signals, filter environmental noise, and reduce operator dependence, creating opportunities for resource-limited malaria screening. However, the majority of these applications have focused on blood-based invasive methods, while noninvasive methods, including VOC analysis,^71,72,74,75^ optical and infrared spectroscopy,^73^ wearable physiological monitoring,^86,88,91^ and mobile imaging,^94^ have seen limited adoption (Table 5).

**Table 5:** Summary of studies on AI-enabled detection and prediction models for noninvasive malaria detection

Machine learning algorithms	Detection method	Sample type	Target biomarkers/features	Diagnostic task	AI contribution	Accuracy	Dataset size	Validation strategy	Infection stage	Ref.
Support vector machine (SVM), k-nearest neighbors (KNN) with cross-validation (CV)	Gas chromatography-mass spectrometry (GC-MS)	Exhaled breath (volatile organic compound (VOC))	9 VOCs (especially, 4 thioethers)	Distinguish infected *vs.* uninfected; correlate VOCs with parasitemia	Perfect separation in cross-validation	Coefficient of determination (*r*^2^) > 0.9	*n* ≈ 50 (breath samples)	Cross-validation (CV; 100%)	Symptomatic & experimental infection	71
Correlation-based feature selection + Nearest Mean Classifier	GC-MS	Exhaled breath (VOC)	6 VOCs + terpenes	Distinguish malaria-positive *vs.* negative children	Identified malaria-specific breathprint	83% overall (specificity: 94%, sensitivity: 71%)	*n* = 35 children	Leave-one-out CV	Pediatric, symptomatic	72
Logistic Regression (Naïve Bayes principle, SoftMax)	Wearable sensors	Vital signs (heart rate (HR), temperature, blood glucose)	Hypoglycemia (<3 mM), fever, heart rate	Predict malaria incidence using noninvasive vital signs	High correlations *r* = 0.99 (glucose), *r* = 0.95 (heart rate)	High predictive accuracy (correlation with malaria incidence)	Case-control data (Ghana, adults)	Not specified	Symptomatic	86
SVM, neural network (NN), KNN, Naïve Bayes	Wearable internet of things (IoT) sensors	Symptom-based physiological signals	15 features (fever, chills, fatigue, anemia, convulsions, *etc.*)	Early detection & classification (parasitized *vs.* normal)	Compared multiple ML models	SVM (best): training 98%, test 96% (area under curve (AUC) 95%)	Simulated IoT dataset	Training/test split (70/30)	Both symptomatic & asymptomatic simulated	91
Adaptive Boosting, random forest (RF), Regularized RF (with recursive feature elimination (RFE))	GC-MS	Skin-emitted VOCs (arm & foot)	Multiple VOCs (toluene, hexanal, nonanal, *etc.*)	Detect symptomatic, asymptomatic, and submicroscopic infections	Identified minimal predictive VOC sets	Sensitivity: 100% for asymptomatic; sensitivity: ∼95%, accuracy: 77–80% overall	*n* ≈ 400 VOC samples	Recursive feature elimination + machine learning (ML)	Symptomatic & asymptomatic	75
Genetic Algorithm + discriminant analysis of principal components (DAPC) & feature selection	GC-MS	Skin volatiles (arm & foot)	VOCs (hexanal, decane, octanal, *etc.*)	Differentiate malaria-positive *vs.* negative (symptomatic children)	Identified malaria-specific VOCs despite overlapping symptoms	100% accuracy in febrile children; ∼75% in mixed-symptom	*n* = 65 children	Feature selection + DAPC	Symptomatic	74
NN (compared with SVM, KNN, RF)	Near-infrared (NIR) spectrometer	Skin (ear, arm, finger)	NIR absorption peaks (937–1660 nm) linked to hemozoin, hemoglobin, parasite proteins	Detect malaria infection (*P. falciparum*, *P. vivax*) across parasitemia levels	NN (best) robust at low parasitemia	Accuracy: 92%, sensitivity: 100%, specificity: 85% (ear spectra)	*n* = 60 subjects (27 malaria-positive: 20 *P. vivax*, 7 *P. falciparum*; 33 malaria-negative); multiple spectra per subject	Training/test comparison	Symptomatic & low parasitemia	73
Neural network classifier (radiomics + mask region-based convolutional neural network (Mask R-CNN) + RF)	Mobile imaging	Smartphone conjunctiva photos	Radiomic features (intensity, texture, transform, radial)	Malaria risk stratification in asymptomatic children	Radiomics + neural network classification	AUC = 0.76 (95% CI: 0.68–0.84)	*n* = 405 children, 4302 photos	Train/test split (70/30)	Asymptomatic	94
Bayesian-based probability model (*2B-Healthy*)	Wearable sensors	Physiological signals (HR, skin temp, actigraphy)	Aberrant HR, activity patterns	Early malaria infection detection in controlled human malaria infection (CHMI) study	Bayesian personalized baseline comparison	Sensitivity: 78%, specificity: 75%; detected ∼6.4 days before parasitemia	*n* = 31 volunteers, CHMI	CHMI study design	Pre-symptomatic detection	88

Traditional and ensemble machine learning have been applied to noninvasive malaria detection. VOC-based GC-MS analyses using SVM, KNN, and nearest mean classifiers demonstrated effective classification of malaria-positive and negative cases, with accuracies ranging from 83% to perfect discrimination and strong correlations with parasitemia.^71,72^ Logistic regression models applied to wearable vital signs achieved near-perfect predictive accuracy (*r* = 0.99 for glucose, *r* = 0.95 for heart rate),^86^ while IoT-based wearables tested multiple classifiers, with SVMs consistently outperforming NN, KNN, and naïve Bayes (testing accuracy up to 96%).^91^ Ensemble approaches, including boosting, random forests, and genetic algorithms, further enhanced detection robustness by identifying minimal VOC feature sets predictive of infection. These methods reached 100% sensitivity for asymptomatic and submicroscopic cases or 100% accuracy in febrile children, though performance in mixed-symptom groups was lower (∼75%).^74,75^ Together, traditional and ensemble machine-learning approaches underscore the value of feature selection and robust classifiers for extracting informative patterns from heterogeneous noninvasive signals.

Deep learning and probabilistic models have extended these capabilities to spectral, imaging, and longitudinal wearable data. Neural networks trained on near-infrared spectra achieved 92% accuracy and perfect sensitivity at very low parasitemia,^73^ while radiomic neural network models applied to smartphone conjunctiva images reached an AUC of 0.76 across diverse devices and participants.^94^ These deep learning methods effectively capture nonlinear relationships and subtle biomarker signatures invisible to human observers. In contrast, probabilistic Bayesian modeling has been leveraged for continuous wearable data, detecting infections with 78% sensitivity and providing early warnings ∼6.4 days before parasitemia or symptom onset.^88^

Across these studies, model choice is closely linked to input data type. VOC studies typically use feature-selected chemical abundance matrices with nearest mean, SVM, random forest, adaptive boosting, or genetic algorithm-based classifiers; spectroscopy studies use wavelength-resolved optical features with neural networks or related classifiers; wearable studies use longitudinal physiological signals and personalized Bayesian or conventional classifiers; and smartphone imaging studies use radiomic or deep image features from conjunctiva photographs. Validation strategies remain heterogeneous, including leave-one-out cross-validation, train/test splits, controlled human infection cohorts, and single-site field cohorts, with relatively limited external validation across countries, devices, age groups, and transmission settings. Reported metrics need cautious interpretation because high cross-validated accuracy in small VOC or simulated wearable datasets may not translate directly to deployment performance in rural field programs with comorbid febrile illness, changing environmental conditions, or different device hardware.

AI algorithms can be tailored to the distinctive characteristics of noninvasive signals, enhancing weak biomarker patterns, reducing environmental noise, and standardizing interpretation. Automated analysis minimizes operator dependence, ensures consistent results in resource-limited settings, and improves sensitivity in cases of low parasitemia or low biomarker concentration. However, broader adoption is hindered by the limited availability of large, diverse, and well-annotated datasets;^71–75,88,91,94^ performance variability due to factors, including calibration drift in optical spectroscopy,^73^ humidity effects on VOC sensors,^71,72,74,75^ and motion artifacts in wearable systems;^88,91^ and the absence of standardized acquisition and interpretation protocols.^71,72,74,75,86,88,91,94^ To address these issues, strategies include creating regionally distributed datasets with standardized acquisition parameters,^71,72,74,75,88,94^ implementing simple on-site calibration routines, including optical target cards or VOC baseline captures,^71–75^ and developing noise-resilient preprocessing pipelines.^71,72,74,75,88,91^ Integrating multimodal data streams that combine chemical, optical, and physiological signals can enhance robustness,^73,88,91,94^ while lightweight AI architectures capable of offline operation are essential for rural deployment.^144–147^

**Fig. 10:**
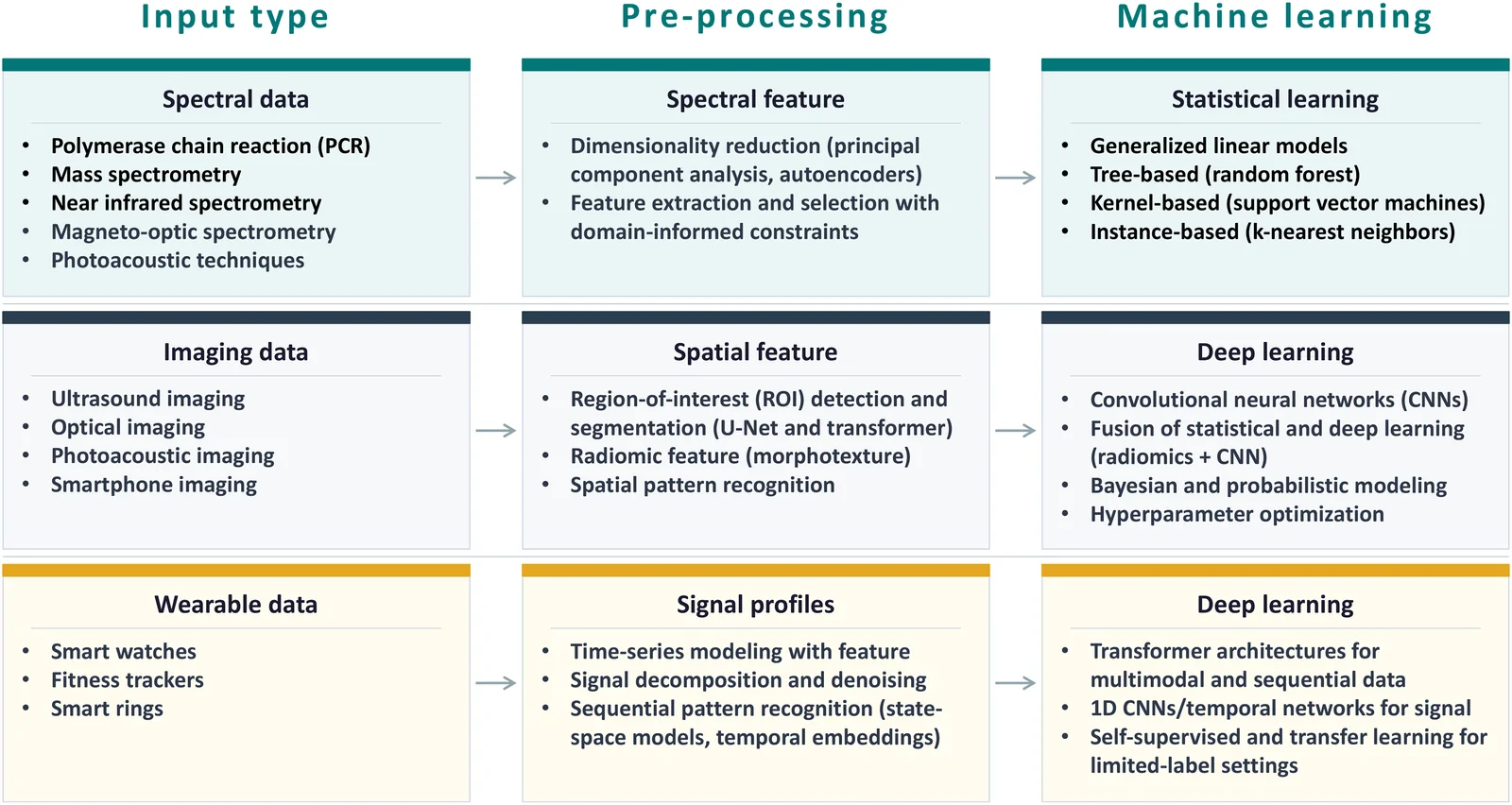
Modality-specific analytical workflows across the three principal domains of noninvasive malaria detection reviewed in this study: biological sample-based methods, acoustic, photoacoustic, and optical systems, and digital and connected health platforms. Original illustration created by the authors.

Overall, Fig. 10 summarizes the modality-specific machine learning workflows across the three principal domains of noninvasive malaria detection. Within each modality, the analytical workflow follows a structured continuum encompassing data acquisition, preprocessing, feature extraction, and model-based interpretation. This workflow highlights that, while a common analytical framework is shared across modalities, the specific implementation of preprocessing, feature representation, and model selection is inherently tailored to the characteristics of each data modality. This modality-specific adaptation is critical for ensuring both analytical performance and translational feasibility across diverse noninvasive malaria detection platforms. Importantly, detection performance is inseparable from translational feasibility. Reliability depends not only on feature quality and model selection, but also on practical constraints (*e.g.*, low biomarker abundance, calibration drift, environmental instability, device variability, infrastructure limitations, and cross-device generalizability).

### 3.4. Experimental platforms for noninvasive malaria detection prior to human applications

**Fig. 11:**
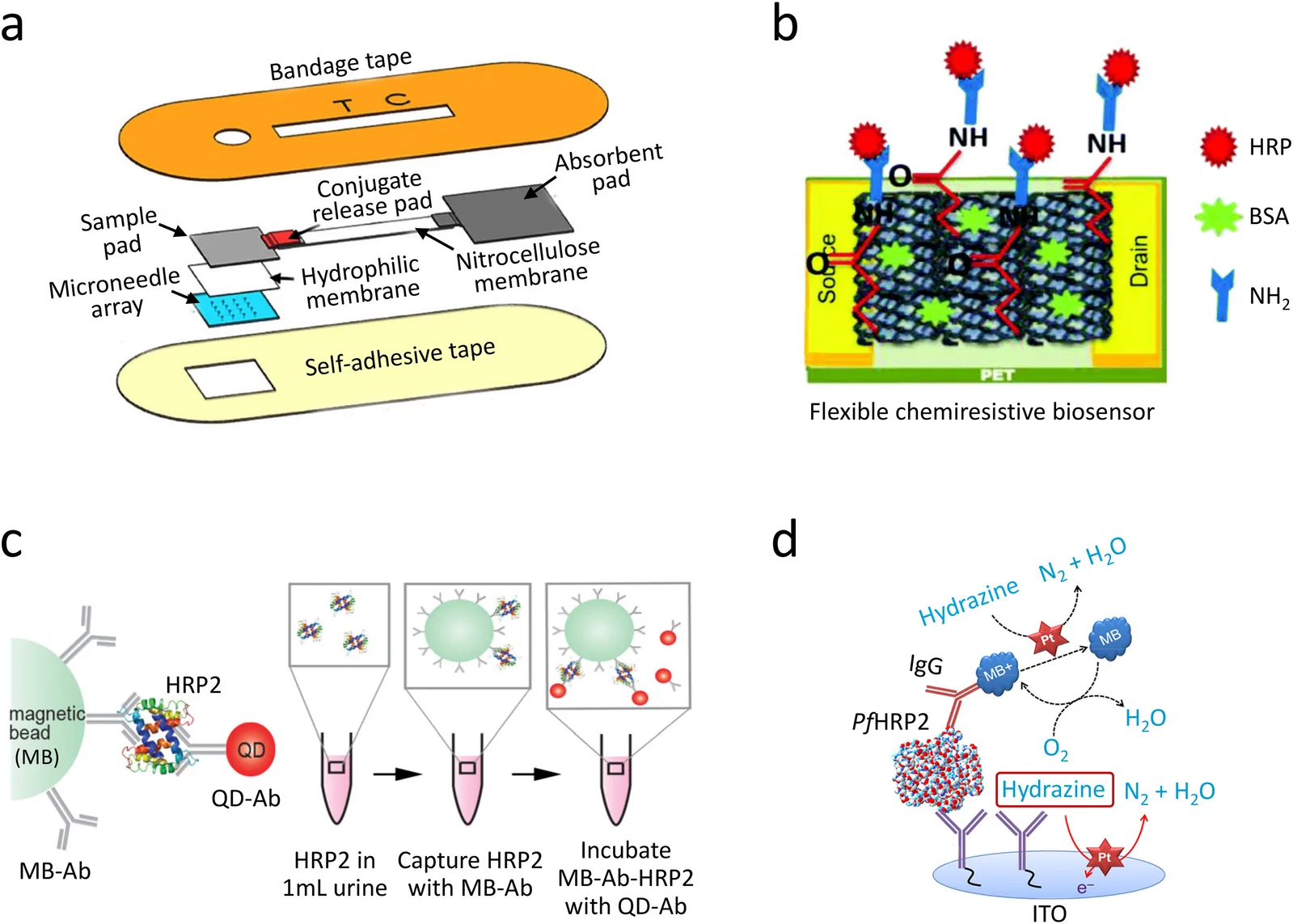
Representative biological sample-based experimental platforms for noninvasive malaria detection prior to human applications. (a) Microneedle-based skin patch for a malaria immunoassay-based strip.^95^ Schematic of the patch showing the microneedle array and integrated lateral flow immunoassay [adapted from ref. 95 under the terms of the CC BY 4.0 license; © 2020 The Author(s).]. (b) Flexible chemiresistive biosensor based on multi walled carbon nanotube (MWCNT)-zinc oxide (ZnO) nanofiber for *P. falciparum* histidine-rich protein 2 (PfHRP2) detection^99^ [adapted from ref. 99 with permission from the Royal Society of Chemistry; © 2017 The Royal Society of Chemistry.]. (c) Nanoparticle-based biosensor assay using magnetic bead (MB) capture and quantum dot (QD) labeling for urinary PfHRP2^97^ [adapted from ref. 97 with permission from the American Society of Tropical Medicine and Hygiene; © 2016 American Society of Tropical Medicine and Hygiene.]. (d) Enzyme-free electrochemical biosensor employing a methylene blue (MB)-labeled immunoglobulin G (IgG) detection antibody and hydrazine oxidation on platinum (Pt) nanoparticles^98^ [adapted from ref. 98 with permission from Elsevier; © 2017 Elsevier].

Noninvasive malaria detection methods have also been reported in animal models or controlled lab simulations before progressing to human studies (Table 6). Biofluid and chemical-sensing platforms have demonstrated a range of advanced strategies. Jiang *et al.*^95^ designed a microneedle-based skin patch that detected PfHRP2 in dermal interstitial fluid at concentrations as low as 8 ng mL^−1^ within 20 minutes, validated in porcine skin as a proof-of-concept blood-free platform (Fig. 11a). Modak *et al.*^96^ developed a saliva-based LAMP assay targeting the *P. falciparum* cytochrome oxidase subunit 1 gene, achieving rapid detection with sensitivity down to ∼1–2 parasites per μL. Biosensors using nanomaterial chemiresistors (limit of detection (LOD) ∼0.97 fg mL^−1^),^99^ quantum dot (QD)-magnetic bead assays (LOD ∼0.5 ng mL^−1^ in urine/serum),^98^ and enzyme-free electrochemical immunosensors (LOD ∼2.8 pg mL^−1^ in saliva)^98^ achieved pg–fg mL^−1^ sensitivity for PfHRP2 detection in spiked saliva or urine (Fig. 11b–d). Capuano *et al.*^100^ further extended detection to infection-associated volatile organic compounds, where quartz microbalance sensor arrays discriminated infected from uninfected mice with ∼80–86% accuracy.

**Table 6:** Summary of preclinical studies on experimental platforms for noninvasive malaria detection

Sample type	Biomarker	Platform details	Performance (sensitivity/specificity/accuracy)	Detection time	Low-resource field usability	Research target	Research area	Detect asymptomatic infection	Ref.
Dermal (skin) interstitial fluid	PfHRP2	Microneedle array-based strips	Limit of detection (LOD): 8 ng mL^−1^ PfHRP2; ∼3 × higher sensitivity than current RDTs; no cross-reactivity with *P. falciparum* lactate dehydrogenase (PfLDH) or aldolase	<20 min	High	Young pig cadavers (proof-of-concept)	USA (lab study)	No	95
Saliva	Mitochondrial cytochrome oxidase subunit 1 gene	Loop-mediated isothermal amplification (LAMP)	Detection limit ∼1–2 parasites μL^−1^; high specificity for *P. falciparum*; can differentiate from *P. vivax*	∼30 min	High	Spiked saliva samples (*P. falciparum* deoxyribonucleic acid (DNA) from reference strains)	USA (lab-based)	Potentially yes (LOD compatible with low parasitemia)	96
Urine	PfHRP2	Magnetic bead-quantum dot (MB-QD) biosensors	LOD 0.5 ng mL^−1^; quantification range 33–2000 ng mL^−1^	NP^a^	Moderate	NP	USA (lab-based)	NP	97
Saliva	PfHRP2	Enzyme-free electrochemical immuno-biosensors	LOD 2.2 pg mL^−1^; linear range 1 pg mL^−1^–100 ng mL^−1^	∼60 min	Moderate	Healthy adults (saliva spiked with recombinant PfHRP2)	NP	NP	98
Nanoparticles (lab-prepared histidine-rich protein 2 (HRP2) solutions)	PfHRP2	Flexible chemiresistive immune-biosensor	Detection limit 0.97 fg mL^−1^; specificity > 95% against non-specific proteins; accuracy NP	∼30 min	Moderate	Lab-prepared HRP2 solutions	India–USA (lab study)	NP	99
Whole-body volatile organic compounds (VOCs) from *Plasmodium berghei*-infected mice	VOCs (nonanal, decanal, and 2-nitro-1,4-benzenedicarboxamide)	Gas chromatography-mass spectrometry (GC-MS)	Cross-validation: 91%/75%/86%; independent test: 84%/73%/81%	∼60 min	Moderate	Female cluster of differentiation 1 (CD1) mice (6–8 weeks) experimentally infected with *Plasmodium berghei* (*P. berghei*)	Italy	No	100
Buccal mucosa; preliminary imaging in human lip	Hemozoin	Portable microvascular microscope: light-emitting diode (LED) illumination (525 nm cross-polarized epi-illumination, 660 nm transmission red light illumination, 550 nm transmission green light illumination)	Detected hemozoin ≥0.03% parasitemia; no false positives; quantitative correlation with parasitemia; accuracy NP	<30 s	Moderate	*Plasmodium yoelii* (*P. yoelii*)-infected mice (*n* = 8) + controls (*n* = 3); healthy human volunteer lip imaged for feasibility	USA (*in vivo* mouse model study)	NP	101
Tissue-simulating phantoms	Hemozoin	Quantitative diffuse optical spectroscopy (DOS): 600–1000 nm broadband NIR	Detection limit 0.014 μg mL^−1^; quantification 0.12 μg mL^−1^; detected 0.16 μg mL^−1^ hemozoin (≈0.5% parasitemia) across 70–90% oxygen (O_2_); accuracy NP	∼10 min	Low	Phantoms with bovine blood (simulating adipose tissue)	USA (lab study)	NP	102
*In vitro*: *P. falciparum* red blood cell (RBC) cultures; *in vivo*: *P. berghei* mice	*In vitro*: parasite byproducts (hemozoin, stage-specific metabolites); *in vivo*: hemozoin pigment and tissue spectral changes	*in vitro*: near-infrared spectroscopy (NIRS) (500–2350 nm) + NN classifier; *in vivo*: NIRS LabSpec 4i, probe on ear/foot/groin/tail	*In vitro*: up to 99.6%/100%/99.6%, LOD <10^−7^ parasites per μL; stage differentiation 98–100%; *in vivo*: 42–94%/79–87%, best ≥3% parasitemia	*in vitro*: 5 sec per spectrum; *in vivo*: ∼35 s per scan	High	*P. falciparum* lab cultures (multi-donor RBCs); Swiss Antigen Reference Centre (ARC) mice (*n* ≈ 144)	Australia	Yes, based on LOD and authors' statement	103

a NP: not provided.

**Fig. 12:**
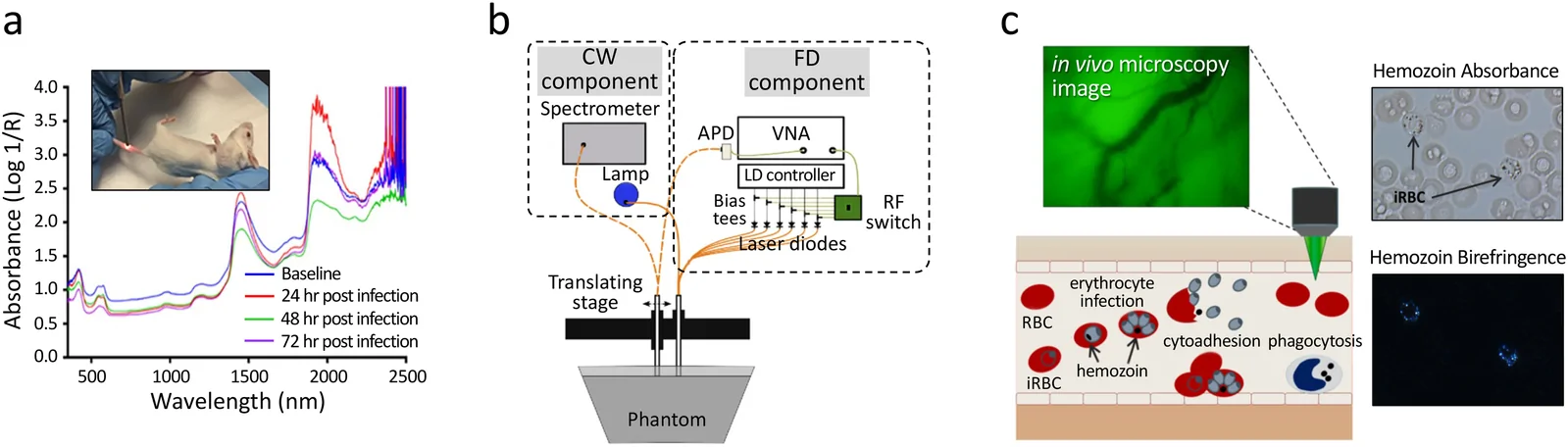
Representative optical and spectroscopic experimental platforms for noninvasive malaria detection prior to human applications. (a) Near-infrared (NIR) spectrometer-based malaria detection with machine learning analysis; representative *in vivo* spectra acquired from mice at baseline and 24, 48, and 72 h post infection.^103^ Inset: mouse scanned with an NIR spectrometer [adapted from ref. 103 under the terms of the CC BY 4.0 license; © 2024 Sikulu-Lord *et al.*]. (b) Combined continuous-wave (CW) and frequency-domain (FD) diffuse optical spectroscopy (DOS) system for hemozoin detection in tissue phantoms.^102^ VNA, vector network analyzer; APD, avalanche photodiode; RF, radio frequency [adapted from ref. 102; © 2020 Optical Society of America under the terms of the OSA Open Access Publishing Agreement.]. (c) *In vivo* microscopy for detecting intracellular hemozoin structures.^101^ Left: Concept of detecting infected cells circulating in superficial microvasculature. Right: Hemozoin visualized under bright-field (top) and cross-polarized illumination (bottom) [adapted with permission from ref. 101; © 2015 Optical Society of America].

Optical and spectroscopic methods have focused on parasite pigment and tissue signatures. Sikulu-Lord *et al.*^103^ demonstrated that near-infrared spectroscopy combined with machine learning detected *P. falciparum* at densities as low as 10^−7^ parasites per μL with 96% sensitivity and 93% specificity, and differentiated asexual stages with ∼98% accuracy *in vitro*. In *Plasmodium berghei* (*P. berghei*)-infected mice, noninvasive scanning of ears or feet achieved 90–94% sensitivity at parasitemia ≥3%, though performance declined markedly below 0.5% (Fig. 12a). Campbell *et al.*^102^ reported that quantitative diffuse optical spectroscopy detected synthetic hemozoin at 0.014 μg mL^−1^ in tissue phantoms, corresponding to ∼0.5% parasitemia, supporting the feasibility of deep-tissue pigment detection despite hemoglobin interference (Fig. 12b). Burnett *et al.*^101^ employed microvascular microscopy to visualize hemozoin absorbance in the buccal mucosa of *Plasmodium yoelii* (*P. yoelii*)-infected mice, successfully detecting crystals across 0.03–50% parasitemia within ∼30 seconds, and further demonstrated imaging feasibility in superficial human lip vasculature (Fig. 12c).

These preclinical studies provide a safe and reproducible environment to validate sensing principles, optimize sampling interfaces, and refine assay chemistries, avoiding the ethical risks of early human testing.^95,96,100–103^ They enable systematic evaluation of parasite density thresholds, co-infections, and host variability under controlled conditions.^102,103^ By assessing detection feasibility across diverse biological sources, including dermal interstitial fluid, saliva, urine, exhaled breath, and optically accessible tissue,^95–101^ these studies identify performance bottlenecks and guide device optimization for field deployment. However, translating to human applications encounters challenges, including variability in human skin structure and immune responses, environmental constraints in endemic regions (*e.g.*, high temperatures and humidity), and the need for standardized protocols to ensure reproducibility.^100,102,103^

## 4. Outlook

Ratings (low, moderate, or high) are based on a synthesis of reported analytical performance, field applicability, cost considerations, and scalability across the reviewed literature, and are not intended as precise quantitative scores. PCR: polymerase chain reaction, LAMP: loop-mediated isothermal amplification, NASBA: nucleic acid sequence-based amplification, GC-MS: gas chromatography-mass spectrometry, VOC: volatile organic compound, pfhrp2/pfhrp3: genes encoding histidine-rich proteins 2 and 3 in *P. falciparum*.

### 4.1. Qualitative comparisons across key implementation dimensions

The noninvasive malaria detection technologies discussed in sections 3.1–3.4 vary substantially in analytical performance, implementation requirements, and translational readiness. The noninvasive malaria detection technologies can be evaluated against established performance requirements for malaria diagnostics in both case management and surveillance settings, including sensitivity, specificity, stability, reliability, and ease of use.^154,155^Table 7 provides a qualitative overview of each technology across key implementation dimensions. Technologies are assessed using semi-quantitative ratings (low, moderate, or high) based on core criteria, including analytical sensitivity, field readiness, cost, and scalability, together with additional attributes such as the ability to detect low-density infections, resilience to *pfhrp2/pfhrp3* gene deletions, and capacity for species differentiation. This framework enables structured comparison while avoiding overinterpretation of heterogeneous evidence from diverse studies and settings. Clear trade-offs emerge across key implementation dimensions: sensitivity for low-density infections is highest with nucleic acid amplification methods, whereas field readiness, cost, and scalability are strongest with immunoassay-based strip tests.

**Table 7:** Qualitative comparison of noninvasive malaria detections across malaria program stages and key implementation dimensions

Detection technology		Sensitivity	Field-readiness	Cost	Scalability	Low density infection	Robustness to *hrp2/3* deletions	Species differentiation
Biological sample-based methods	Immunoassay-based strips	Moderate	High	Low	High	Low–moderate	Low (*hrp2/3*-dependent)	Low
	PCR/LAMP/NASBA	High	Low–moderate	High	Moderate	High	High (*hrp2/3*-independent)	High
	Biosensors	High (analytical)/Moderate (clinical)	Low	Moderate–high	Low	Moderate	Low (*hrp2/3*-dependent)	Moderate
	VOC-based GC-MS	High (selected studies)	Low	High	Low	Moderate	High	Low–moderate
Acoustic/photoacoustic/optical systems	Ultrasound	Low (non-parasitological)	Moderate–high	Moderate	Moderate	Low	High (*hrp2/3*-independent)	None
	Photoacoustic	Moderate–high	Low	High	Low	Moderate	High (*hrp2/3*-independent)	Low
	Optical spectroscopy/imaging	Moderate	Moderate	Moderate	Moderate	Low–moderate	High (*hrp2/3*-independent)	Low–moderate
Digital and connected platforms	Wearable devices	Low–moderate	Low–moderate	Moderate	Low	Low	High (*hrp2/3*-independent)	None
	Mobile imaging	Moderate	High	Low	High	Low–moderate	High (*hrp2/3*-independent)	Low

Immunoassay-based strip tests are characterized by high field readiness, low cost, and strong scalability, but show reduced sensitivity for low-density infections and vulnerability to hrp2/3 gene deletions with limited capacity for species differentiation. By contrast, nucleic acid amplification methods offer superior analytical sensitivity for submicroscopic and asymptomatic infections, while also showing robustness to gene deletions and strong species-level identification capability, albeit at the cost of greater infrastructure and operational complexity. Biosensors and VOC-based approaches have reported encouraging analytical performance, but their broader application remains constrained by limited field readiness, challenges in standardization, and, in some cases, dependence on HRP2/3-related biomarkers or variable metabolic signatures that may reduce robustness across settings. Acoustic, photoacoustic, and optical modalities, including ultrasound, photoacoustic techniques, and optical spectroscopy, are generally independent of HRP2/3 biomarkers and are not affected by *hrp2/3* gene deletions. However, their diagnostic role differs by modality: ultrasound detects organ-level or physiological changes rather than parasites directly, whereas photoacoustic and optical spectroscopy approaches may target hemozoin or spectral signatures more closely linked to infection. As a result, sensitivity for low-density infections and species differentiation remain limited, especially for ultrasound and other indirect physiological readouts. Digital and connected platforms, such as wearable devices and smartphone-based imaging, provide high scalability and accessibility, but depend on indirect physiological or visual signals, resulting in lower specificity, limited sensitivity for low-density infections, and little to no species differentiation.

### 4.2. Limitations and sources of variability in current malaria detection using nonblood sampling

#### 4.2.1. General limitations and variabilities

Several key biological, technical, and operational factors contribute to variability and limitations in noninvasive malaria detection. First, confounding biological and physiological factors influence biomarker presence and detectability in nonblood samples.^2,106^ Unlike parasite density in blood, biomarkers in urine, saliva, or breath are subject to dynamic fluctuations. Their concentrations may vary with circadian rhythms, hydration status, and renal or salivary clearance, leading to temporal inconsistency in detection signals.^104–106^ Second, detection sensitivity is significantly reduced at low parasite density (*e.g.*, ≤200 parasites per μL), which is particularly problematic in asymptomatic infections that contribute disproportionately to malaria transmission in near-elimination settings.^5,47,48,156^ Third, genetic variability across *Plasmodium* species, including *hrp2/3* gene deletions, can lead to false-negative results in antigen-based assays, highlighting the challenge of developing universally reliable detection platforms.^157^ Fourth, the presence of co-infections and mixed-species infections may further alter biomarker expression or introduce confounding signals, reducing detection specificity.^158^ These factors limit robustness and field applicability, particularly under tropical conditions and in the detection of asymptomatic low-density infections, as well as in the context of co-infections and *Plasmodium* species diversity. Finally, field deployment in endemic regions is complicated by high ambient temperatures and limited infrastructure, which can accelerate biomarker degradation and compromise sample stability during storage and transport.^44^ These factors limit robustness, reproducibility, and field applicability, particularly under tropical conditions, in asymptomatic low-density infections, and in the context of co-infections and *Plasmodium* species diversity.^106,156^

#### 4.2.2. Modality-specific limitations

In acoustic, photoacoustic, and optical methods, sensitivity and specificity may be compromised by operator dependence, skin pigmentation, motion artifacts, device calibration, and the requirement for specialized instrumentation and training, limiting validation across diverse populations.^26,73,77,79,81^ VOC-based detection approaches are influenced by host metabolism, diet, microbiome composition, and environmental exposure, which can reduce specificity.^71,110^ In addition, VOC profiles are sensitive to environmental conditions (*e.g.*, temperature and humidity) and variability in sampling and a lack of standardized collection protocols can limit reproducibility.^110^ Moreover, VOC analysis often relies on complex instrumentation (*e.g.*, GC-MS), constraining scalability and field deployment.^159^ Buccal mucosa-based detection also presents challenges, including low biomarker abundance, potential contamination from oral microbiota and debris, and variability in swab collection techniques, with limited clinical validation compared to other noninvasive sample types.^160,161^ Digital and connected platforms are not designed to detect and differentiate all parasite species.^88,94^ In some endemic settings, while the minimum requirement is detection of *P. falciparum* and/or *P. vivax*, species differentiation is highly desirable. These platforms also rely heavily on signal quality and robust model training, which introduces challenges related to generalizability and regulatory approval. In addition, their implementation remains constrained by limited device penetration and infrastructure in many endemic regions.^86,124,127,142^ These limitations highlight the importance of integrating digital approaches with conventional malaria detection methods. Table 8 summarizes the key features, advantages, and limitations of the three principal categories of noninvasive malaria detection discussed in this review.

**Table 8:** Comparative summary of noninvasive malaria detection methods across three major categories

	Key features	Advantages	Limitations
Biological sample-based methods	Immunoassay-based strips	Painless, culturally acceptable sampling; may improve participation among vulnerable groups (*e.g.*, children, women)	Low biomarker concentration compared to blood
	Nucleic acid amplification (polymerase chain reaction (PCR), loop-mediated isothermal amplification (LAMP), nucleic acid sequence-based amplification (NASBA))	Compatible with immunoassay-based strips/molecular tests, high sensitivity, multi-biomarker use	Potential interference from sample contaminants
	Biosensors	Supports multi-biomarker detection	Variable sensitivity and reproducibility; lack of standardized protocols
	Gas chromatography-mass spectrometry (GC-MS)	Portable and suitable for low-resource settings	Biomarker variability influenced by hydration status, circadian rhythms, and parasite sequestration
			Challenges in detecting low-density or asymptomatic infections and maintaining sample stability under field conditions
Acoustic, photoacoustic, and optical systems	Ultrasound (transcranial Doppler (TCD), optic nerve sheath diameter (ONSD), abdominal)	Fully noninvasive; no sample collection required, reducing barriers to repeated testing	Sensitivity to skin tone and motion artifacts, potentially affecting performance across populations
	Photoacoustic (imaging, spectroscopy)	Label-free detection without reagents or consumables	Requires specialized instrumentation and hardware
	Optical spectroscopy (near-infrared (NIR), Raman, fluorescence, diffuse)	Real-time, *in vivo* monitoring of infection-related changes	Limited validation in diverse populations, often operator dependent
			Limited sensitivity at low parasitemia and variability in host physiological signals
Digital and connected platforms	Wearable physiological signal monitoring	Scalable for decentralized, community-based surveillance	Dependence on signal quality and environmental factors
	Mobile imaging	Enables individualized, automated, continuous monitoring	Requires model training and validation
	Artificial intelligence (AI)-enabled analytics	Portable and suitable for low-resource field settings using existing consumer devices	Generalizability concerns and limited device access in endemic regions
			Model performance may be affected by population heterogeneity, co-infections, and limited representative datasets

### 4.3. Context-specific utility and equity

The optimal deployment of noninvasive malaria detection methods depends on context, particularly in relation to infrastructure, resource availability, and stage-specific programmatic needs. Acoustic, photoacoustic, and optical tools may be advantageous where consumables are limited but trained operators, maintenance, and hardware support are available, whereas digital platforms enable decentralized surveillance and outbreak monitoring but raise challenges in equitable access. Increasingly, hybrid strategies, such as combining volatile biomarker profiling with wearable sensors or integrating spectroscopy with AI-enabled analytics, are being explored to enhance diagnostic performance and operational adaptability across settings.^100,103,124^ Thus, translation is constrained by both intrinsic analytical bottlenecks and system-level implementation challenges. Analytical limitations include low biomarker abundance, indirect physiological signals, environmental interference, and limited external validation; implementation barriers include affordability, training, maintenance, connectivity, supply chains, and integration into care pathways. Beyond technical deployment considerations, equitable implementation of noninvasive malaria detection requires addressing broader issues of access, infrastructure, and population diversity.^8,14^ Regional datasets and multi-country validation are needed to avoid bias in algorithm development and device calibration. Advances in sensor design, end-to-end wearable platforms,^126^ and smartphone-based optical biosensors^124,129^ provide pathways toward scalable, low-cost systems. However, disparities in digital infrastructure, device availability, and data quality across settings may affect real-world performance and deployment consistency, particularly in low-resource environments. Regulatory and technical barriers in such contexts^127^ further highlight the need for adaptive validation and certification frameworks tailored to diverse implementation conditions.

At the community level, successful adoption depends on user acceptability, cultural context, and trust in healthcare systems. The uptake of noninvasive sampling methods and digital platforms is closely shaped by local perceptions and engagement practices.^9,109^ Noninvasive and privacy-preserving approaches may improve participation among women, children, and primary caregivers by reducing barriers associated with blood-based testing. Nevertheless, sustained community engagement and context-specific implementation strategies remain essential to ensure uptake, particularly in resource-limited or geographically isolated regions.^6^ Alignment with World Health Organization (WHO) malaria surveillance priorities and elimination roadmaps will be essential to ensure that technological innovation translates into scalable and inclusive real-world impact.

### 4.4. Considerations in machine learning and AI

A central challenge for AI-enabled and digital detection is generalizability: model performance often degrades when models are applied to new sites, devices, environmental conditions, or populations.^71–75^ Most studies rely on relatively small, geographically narrow and modality-specific datasets, often collected under heterogeneous conditions and linked to variable reference standards. These limitations are especially important in resource-limited settings, where low-density or asymptomatic infections, inconsistent annotation quality, device variability and fragmented infrastructure can exacerbate domain shift and undermine generalizability. Progress will require not only larger datasets, but also prospectively curated, regionally distributed cohorts, standardized acquisition protocols, site-aware validation strategies and data-efficient learning approaches, including transfer learning, weak supervision, multimodal fusion and lightweight models suitable for offline or low-connectivity use. Future work should also consider domain adaptation, synthetic augmentation, and cross-site/device training, as well as privacy-preserving federated learning to enable multi-country updates without centralizing data.^144^

Emerging guidance for trustworthy clinical AI should also be considered, including the Transparent Reporting of a multivariable prediction model for Individual Prognosis Or Diagnosis-Artificial Intelligence (TRIPOD-AI), Developmental and Exploratory Clinical Investigation of Decision-support systems driven by Artificial Intelligence (DECIDE-AI), and Fairness, Universality, Traceability, Usability, Robustness, and Explainability of AI (FUTURE-AI) frameworks.^145–147^ Future studies should report not only discrimination metrics, such as area under the curve, sensitivity, and specificity, but also calibration, external validation across sites and devices, robustness to environmental variation, subgroup performance, handling of missing data, and prospective utility under realistic workflow conditions. Whenever possible, evaluation should extend beyond random data splits to patient-level, site-level, device-level and temporal validation designs that better reflect real deployment. Lightweight, offline-capable architectures and energy-aware deployment strategies are further needed to ensure robustness in resource-limited field settings.^130^ It is important to recognize the limitations of conventional AI paradigms that rely on massive scale (*e.g.*, extremely large datasets, petabyte-level data storage, hundreds of thousands to millions of graphics processing unit (GPU) hours, and multiple gigawatt-hours of electricity per model). For emerging biomedical engineering technologies to be truly scalable and deployable in resource-limited settings, including environments with constrained cloud computing access, it will be essential to develop efficient machine learning and AI approaches that do not depend on massive training datasets, heavy computational resources, or extremely complex algorithms.^162,163^ Concurrent progress in portable devices, connectivity, and AI-driven modeling points toward scalable solutions for surveillance and case detection.^88,124,125^ Edge (on-device) AI, enabled by pruning, quantization, and distillation, represents a particularly practical direction for translational biomedical engineering platforms for noninvasive malaria detection.

### 4.5. Bridging research and ensuring scalability

The critical next step is bridging proof-of-concept studies and reliable field deployment. Translation requires feasibility, affordability, and alignment with global health implementation frameworks. Challenges include environmental variability,^8^ asymptomatic parasitemia,^4,5,14^ and risk of resurgence in high-burden regions,^1,6^ which demand quality-control, reproducible calibration, and multisite validation.^164^ Scalability further depends on portability, durability, and system integration.^117,126^ Advances in microfluidics, portable spectroscopy, and digital platforms have produced portable proof-of-concept systems,^95,103,104^ while spectroscopy with AI and VOC-profiling studies have reported detection of low-density or asymptomatic infections.^69,91,103^ Ultimately, uptake will hinge on regulatory approval, community acceptance, and alignment with elimination strategies.^124,127,142^ Embedding noninvasive detection within mobile health applications and community workflows could provide sustainable channels for integration into national programs, strengthening case detection and surveillance in rural and underserved areas.^129,142,143^ Addressing these translational steps will determine whether noninvasive detection remains a research concept or becomes a durable component of malaria control and elimination. Finally, integration is essential: standardized AI outputs and digital detection should feed directly into malaria information platforms and national surveillance systems, enabling programmatic relevance.^1,143^

The REASSURED framework can guide development and evaluation of noninvasive malaria detection tests:^167^ real-time connectivity, ease of specimen collection, affordability, sensitivity, specificity, user-friendliness, rapidness and robustness, equipment-free or simple operation, environmental friendliness, and deliverability to end users. A successful test should use an acceptable noninvasive specimen, such as saliva or urine, with standardized collection and minimal sample-processing requirements; achieve sensitivity and specificity appropriate for its intended use against a validated reference standard; and provide an actionable result during the same patient visit. The assay should be simple enough for community health workers or self-testing, remain stable during transport and storage under field temperature and humidity conditions, and require no equipment beyond a low-cost portable reader or smartphone, where needed. Digital result capture could reduce subjective interpretation, support quality assurance, and enable real-time case reporting and surveillance. Finally, evaluation should extend beyond analytical accuracy to include usability, clinical impact, cost-effectiveness, supply-chain feasibility, waste disposal, and equitable delivery to populations with the greatest need.

### 4.6. Regulatory pathways

For noninvasive malaria detection devices, regulatory authorization may provide an important signal of credibility, but it is unlikely to be sufficient, on its own, to enable broad international adoption. In practice, country-specific approval for medical devices or software remains necessary, and the extent to which authorization by the US Food and Drug Administration (FDA) or conformity with European Union (EU) regulatory requirements can facilitate local review varies across jurisdictions. Within the EU, products are regulated under Regulation (EU) 2017/745 on medical devices (MDR) or Regulation (EU) 2017/746 on *in vitro* diagnostic medical devices (IVDR), depending on intended purpose. Software with a medical purpose may fall within the scope of the MDR, whereas platforms intended for *in vitro* examination of specimens may instead be regulated under the IVDR. In both cases, Conformité Européenne (CE) marking follows conformity assessment.

Across Africa, some regulators have introduced reliance mechanisms that may accelerate review for products already authorized in recognized reference markets, whereas others continue to require more extensive national assessment. Uganda, for example, has established a Track 1 pathway for devices approved in International Medical Device Regulators Forum member countries. Ghana has indicated that it may rely on decisions from other regulatory authorities for medical devices, Rwanda may use assessments and audits from recognized regulators or conformity-assessment bodies, including through abridged or expedited review in some circumstances, and South Africa has issued guidance on medical-device reliance.

Regulatory authorization, however, does not by itself resolve the broader policy context of malaria diagnosis. Current World Health Organization guidance continues to recommend confirmation of suspected malaria through parasite-based testing, typically microscopy or rapid diagnostic testing, before treatment. Accordingly, noninvasive devices may have a more tractable initial path to regulatory acceptance and programmatic uptake when positioned as tools for screening, triage or diagnostic support, rather than as direct replacements for microscopy or rapid diagnostic tests. This positioning remains partly interpretive, but is consistent with the current structure of WHO diagnostic policy.

WHO prequalification and product testing programs, developed with international diagnostic partners, can provide an important framework for evaluating noninvasive malaria detection tests.^154^ A further complexity is that WHO prequalification is not equivalent to FDA authorization or CE marking. WHO’s current prequalification pathway for diagnostics is directed towards *in vitro* diagnostic medical devices and excludes medical devices outside that category. Thus, if a noninvasive malaria detection platform does not depend on *ex vivo* specimen testing, it may need to proceed primarily through medical-device or software-as-a-medical-device pathways rather than through conventional malaria *in vitro* diagnostic frameworks. This distinction is particularly important because procurement pathways for malaria diagnostics, including those linked to major global health funders, remain closely aligned with WHO-prequalified products or other mechanisms applicable to appropriately classified *in vitro* diagnostics. More broadly, market access across Africa remains largely country-specific despite ongoing regional harmonization efforts, meaning that successful translation will require attention not only to the product dossier itself, but also to the regulatory roles and responsibilities of the manufacturer, authorized representative, importer and distributor.

## 5. Conclusion

This comprehensive review is intended to initiate discussion of a practical roadmap that links innovation in noninvasive malaria detection to programmatic implementation and aligns these detection strategies with the evolving needs of malaria programs. We synthesize recent biomedical engineering advances in noninvasive malaria detection, beginning with diverse sampling matrices, including urine, saliva, buccal mucosa, exhaled breath, volatile organic compounds, and optically accessible tissue. These sources contain parasite- and host-derived biomarkers that support the development of noninvasive detection strategies. A clear understanding of the underlying biophysical detection mechanisms spanning biological, optical, and digital domains, together with preclinical experimental platforms, helps us to elucidate their complementary strengths and limitations in local implementation settings. Collectively, these advancements illustrate a rapidly expanding landscape of biomedical engineering approaches aimed at extending malaria detection beyond conventional blood-based testing.

The success of noninvasive malaria detection methods lies not only in analytical performance but also in their ability to enhance patient acceptability, enables repeated testing, and expand reach in both clinical and community settings. Such strategies are particularly valuable for underrepresented groups including children and asymptomatic carriers that sustain hidden transmission reservoirs^4,5^ and are generally viewed favorably by end users in endemic countries.^25^ Noninvasive platforms should be considered complementary to blood-based detection, offering safer, more frequent, and culturally acceptable surveillance opportunities in endemic regions, while careful attention to implementation and regulatory challenges will be essential for real-world deployment.^127^ Advancing biomedical engineering platforms for noninvasive malaria detection requires integration into broader health systems and infrastructure strengthening efforts, as long-term sustainability will depend on affordability, regulatory confidence, and alignment with global malaria control, elimination, and eradication strategies.^1^

This review seeks to accelerate progress toward malaria elimination by guiding researchers, engineers, technology developers, diagnostic scientists, clinicians, epidemiologists, public health practitioners, policymakers, regulators, manufacturers, implementation scientists, and community stakeholders toward the most evidence-aligned and equitable solutions. The WHO Global Technical Strategy (GTS) for Malaria 2016–2030 was designed to accelerate progress toward malaria elimination with targets of a 90% reduction in global malaria incidence and mortality and elimination in at least 35 countries by 2030.^165^ However, the Global Malaria Programme operational strategy 2024–2030 underscores that current gains remain insufficient, and that global progress in reducing malaria morbidity and mortality is markedly off track.^166^ Against this backdrop, noninvasive malaria detection strategies enabled by advances in biomedical engineering and machine learning may offer one pathway to support the GTS targets through technological innovation and translational breakthroughs.

## Conflicts of interest

There are no conflicts to declare.

## Supplementary Material

SD-005-D6SD00085A-s001

## Data Availability

No new data were created or analyzed in this study. Data sharing is not applicable to this article. Supplementary information (SI): Table S1 includes a glossary of key terms and abbreviations used in the article. See DOI: https://doi.org/10.1039/d6sd00085a.
