# Supplementary material for: Noninvasive malaria detection beyond blood sampling
Source: Sens Diagn. 2026 Jul 21;5(9):1037–87. doi: 10.1039/d6sd00085a (PMC13463358; doi:10.1039/d6sd00085a)
Supplement: Supplementary file 1 [file SD-005-D6SD00085A-s001.pdf]

Supplementary Information (SI) for Sensors & Diagnostics.
This journal is © The Royal Society of Chemistry 2026

Supplementary Information

Noninvasive malaria detection beyond blood sampling

Shaun G. Hong, Jung Woo Leem, Haripriya Sakthivel, Semin Kwon, Sang Mok Park, Atin Dewan, David J. Botana, and Young L. Kim

Table S1. Glossary of key terms used in the paper

Term	Definition
18S ribosomal RNA (18S rRNA)	A component of the small ribosomal subunit; commonly used as a highly sensitive PCR target for *Plasmodium* detection
Aldehydes	Organic compounds detected in volatile organic compound (VOC) profiles, often associated with oxidative stress
Aldolase	A glycolytic enzyme of *Plasmodium* parasites used as a biomarker for multiple species
Anopheles	A genus of mosquitoes that serves as the vector transmitting malaria parasites to humans
Antigen	A molecule recognized by the immune system, often used as a diagnostic target in malaria tests
Apical membrane antigen 1 (AMA-1)	A *Plasmodium* antigen involved in merozoite invasion of host cells and commonly used as a target in malaria immunoassays and serological studies.
Autochthonous cases	Locally acquired infections occurring within a specific geographic region
Buccal mucosa	The inner lining of the cheek used as a noninvasive sampling site
Circadian variation	Biological fluctuations over a ∼24-hour cycle affecting biomarker levels
Circumsporozoite protein (CSP)	A major surface protein of malaria sporozoites used in vaccines and antibody detection
Conjunctiva	A thin membrane covering the eye, used as a noninvasive site for imaging-based assessment of microvascular and hematological changes
Controlled Human Malaria Infection (CHMI)	A research model in which healthy volunteers are intentionally infected with malaria under controlled conditions to study disease progression, immunity, and interventions
Cytochrome b (*cytb*)	A mitochondrial gene used as a molecular marker for parasite detection
Cytochrome c oxidase subunit 3 (cox3)	A mitochondrial gene target with high copy number that is commonly used in sensitive molecular detection of *Plasmodium*, especially in low-density infections.
Cytophone	A photoacoustic-based flow cytometry technique that detects circulating cells or particles in vivo by measuring laser-induced acoustic signals, often used for noninvasive blood analysis
Droplet digital PCR (ddPCR)	A highly sensitive nucleic acid quantification method that partitions a sample into thousands of droplets for absolute target detection without reliance on standard curves.
Electronic nose (e-nose)	A sensor system designed to detect and classify volatile organic compounds (VOCs)
Enzyme-linked immunosorbent assay (ELISA)	A technique detecting antigens or antibodies via enzyme-mediated signals
Endothelial glycocalyx	A vascular surface layer whose disruption is associated with severe malaria
Gas chromatography-mass spectrometry (GC-MS)	An analytical method for identifying chemical compounds including volatile organic compounds (VOCs)
Gametocyte	A sexual parasite stage responsible for transmission to mosquitoes
Hemozoin	A crystalline byproduct of hemoglobin digestion by malaria parasites
Hepatosplenomegaly	The abnormal enlargement of both the liver and spleen, often associated with systemic infections such as malaria
Hypnozoite	A dormant liver-stage form of *Plasmodium vivax* and *Plasmodium ovale* that can reactivate and cause relapse weeks to months after the initial infection
Immunoassay	A biochemical method detecting antigens or antibodies
Immunoglobulin G (IgG)	A major class of antibodies found in blood and extracellular fluid, involved in immune response and commonly used as a marker of past or ongoing infection
Immunoglobulin M (IgM)	A class of antibodies that is produced early during the initial immune response to infection, indicating recent or acute exposure to a pathogen
Interdigitated electrode (IDE)	A microelectrode structure composed of interlocking conductive fingers, commonly used in impedimetric biosensors to detect analyte-induced electrical changes.
Isothermal amplification	Nucleic acid amplification at constant temperature
Limit of detection (LOD)	The lowest concentration of a target analyte that can be reliably detected by a diagnostic method
Loop-mediated isothermal amplification (LAMP)	A rapid isothermal DNA amplification technique
Lung ultrasound (LUS)	A noninvasive ultrasound-based imaging approach used to assess pulmonary abnormalities such as edema or consolidation, including complications associated with severe malaria.
Mask R-CNN	A deep learning-based instance segmentation model that detects objects in an image while simultaneously generating pixel-level masks for each object

Table S1. Glossary of key terms used in the paper (continued)

**Term**	**Definition**
Merozoite surface protein 1 (MSP1)	A parasite protein used for immune response detection
Messenger RNA (mRNA)	RNA molecule used as a diagnostic target
Middle Cerebral Artery (MCA)	A major artery supplying blood to large portions of the brain, commonly assessed using transcranial Doppler ultrasound to evaluate cerebral blood flow
Near-infrared spectroscopy (NIRS)	A noninvasive optical technique that measures tissue absorption of near-infrared light to assess physiological and biochemical properties
Nested polymerase chain reaction (Nested PCR)	A two-step PCR method designed to improve sensitivity and specificity by using two sets of primers in successive amplification reactions
Optic nerve sheath diameter (ONSD)	A surrogate marker for intracranial pressure
Palpebral conjunctiva	The mucous membrane lining the inner surface of the eyelids, often used as a noninvasive site for assessing anemia or microvascular changes
Parasite lactate dehydrogenase (pLDH)	An enzyme indicating viable malaria parasites
Photoacoustic spectroscopy	A technique that measures acoustic signals generated by the absorption of modulated light, enabling sensitive detection of optical absorption properties in biological samples
Photoacoustic imaging	Imaging based on optical-to-acoustic signal conversion
Plasmodium	A genus of protozoan parasites causing malaria
Plasmodium ovale (*P. ovale*)	A malaria parasite that can cause mild disease and relapse due to dormant liver stages (hypnozoites)
Plasmodium falciparum (*P. falciparum*)	The most severe malaria parasite species
Plasmodium falciparum erythrocyte membrane protein 1 (PfEMP1)	A major variant surface protein expressed on *Plasmodium falciparum*-infected erythrocytes that contributes to cytoadherence and is also used as an immunological marker in malaria studies.
Plasmodium falciparum surface protein 25 (Pfs25)	A gametocyte- or ookinete-associated transcript/protein marker used to assess transmission-stage parasites, particularly in molecular detection assays.
Plasmodium falciparum vacuole membrane protein *(Pfs16)*	A gametocyte-associated transcript marker used in malaria molecular assays to identify transmission-stage parasites.
Plasmodium glutamate dehydrogenase *(pGDH)*	A parasite-derived metabolic enzyme used as a biomarker of active malaria infection and ongoing parasitemia.
Plasmodium malariae (P. malariae)	A human malaria parasite species that typically causes quartan malaria and is generally associated with lower parasitemia than *Plasmodium falciparum*.
Plasmodium knowlesi (*P. knowlesi*)	A zoonotic malaria parasite transmitted from macaques to humans, capable of causing severe infection
Plasmodium vivax (*P. vivax*)	A relapsing malaria parasite species
Plasmodium falciparum Histidine-rich protein 2 (PfHRP2)	A protein from *Plasmodium falciparum* commonly used in RDTs
*pfhrp*2/3 deletion	Genetic deletions of histidine-rich protein 2 and 3 genes in *Plasmodium falciparum*, which can lead to false-negative results in HRP2-based rapid diagnostic tests
Polymerase chain reaction (PCR)	A DNA amplification technique
Quantitative nucleic acid sequence-based amplification (QT-NASBA)	An isothermal RNA amplification technique that enables quantitative detection of nucleic acids, commonly used for detecting low-abundance RNA targets in malaria diagnostics
Quantitative polymerase chain reaction (qPCR)	A polymerase chain reaction method that monitors amplification in real time to quantify nucleic acid targets, commonly using fluorescent readouts.
Radiomics	A method that extracts large numbers of quantitative features from medical images to characterize tissue properties and support data-driven analysis
Renal filtration	Kidney process filtering blood into urine
Reverse transcription (RT)	Conversion of RNA into complementary DNA (cDNA), commonly used prior to amplification in RNA-based diagnostics
Reverse transcription loop-mediated isothermal amplification (RT-LAMP)	RNA amplification under isothermal conditions
Rolling-circle enhanced enzyme activity detection (REEAD)	A biosensing method that detects enzyme activity by generating circular DNA templates followed by rolling circle amplification, enabling highly sensitive readout of targets such as *Plasmodium* topoisomerase I.
Sequestration	The process by which malaria-infected red blood cells adhere to and accumulate in microvasculature, reducing their presence in peripheral circulation
Sporozoite	The infective stage of *Plasmodium* transmitted by mosquitoes to humans
Thioethers	Sulfur-containing volatile organic compounds (VOCs) associated with malaria infection
Topoisomerase I (Top I)	An enzyme involved in DNA replication and a potential biomarker
Transcranial Doppler (TCD)	Ultrasound method measuring cerebral blood flow

Table S1. Glossary of key terms used in the paper (continued)

**Term**	**Definition**
Transdermal detection	Detection through the skin without invasive sampling
Transmission	Spread of parasites from mosquitoes to humans
Var acidic terminal sequence (*varATS*)	A multicopy *Plasmodium falciparum* genomic target used in sensitive nucleic acid amplification assays for malaria detection, especially in low-density infections.
Vector	An organism that transmits pathogens between hosts
Volatile organic compounds (VOCs)	Small gaseous molecules reflecting metabolic processes
